# Proteomics and human microchips identify Thrombospondin-1 as a potential biomarker for calciphylaxis stem cell therapy

**DOI:** 10.1016/j.isci.2026.116388

**Published:** 2026-06-19

**Authors:** Jiaying Hu, Shijiu Lu, Lianju Qin, Yaoting Sun, Xiaoxue Ye, Qinyi Lin, Jing Zhang, Ming Zeng, Jingjing Wu, Kang Liu, Jingfeng Zhu, Ling Zhang, Feng Chen, Zaozao Chen, Shihui Xu, Zhangzhi Xue, Yongwu Yu, Lu Li, Weigang Ge, Zhongze Gu, Cui Li, Zhonglan Su, Dan Luo, Shaowen Tang, Xinfang Tang, Wuziyi Ji, Anning Bian, Meihua Liao, Guicun Fang, Xiang Ma, Song Ning, Yugui Cui, Chunyan Jiang, Huimin Wu, Baiqiao Zhao, Xiuqin Wang, Ningxia Liang, Tingyu Xu, Jiayin Liu, Yun Liu, Tiannan Guo, Yi Zhu, Ningning Wang

**Affiliations:** 1 Division of Nephrology, Department of Geriatrics, the First Affiliated Hospital with Nanjing Medical University, Jiangsu Province Hospital, Nanjing 210029, China; 2 Department of Nephrology, the First Affiliated Hospital with Nanjing Medical University, Jiangsu Province Hospital, Nanjing 210029, China; 3 Department of Nephrology, The Affiliated Huaihai Hospital of Xuzhou Medical University, Xuzhou 221004, China; 4 State Key Laboratory of Reproductive Medicine and Offspring Health, Center of Clinical Reproductive Medicine, the First Affiliated Hospital with Nanjing Medical University, Jiangsu Province Hospital, Nanjing 210029, China; 5 School of Medicine, Westlake University, Hangzhou 310024, China; 6 Westlake Center for Intelligent Proteomics, Westlake Laboratory of Life Sciences and Biomedicine, Hangzhou 310024, China; 7 Research Center for Industries of the Future, School of Life Sciences, Westlake University, Hangzhou 310024, China; 8 Department of Nephrology, Putuo District Central Hospital of Shanghai, Shanghai 200063, China; 9 Department of Nephrology, China-Japan Friendship Hospital, Beijing 100029, China; 10 Department of Epidemiology and Biostatistics, School of Public Health, Nanjing Medical University, Nanjing 211166, China; 11 State Key Laboratory of Digital Medical Engineering, School of Biological Science and Medical Engineering, Southeast University, Nanjing 210096, China; 12 Jiangsu Avatarget Biotechnology Co., Ltd., Nanjing 211100, China; 13 Southeast University Nanjing Jiangbei New Area Innovation Institute, Nanjing 211800, China; 14 Department of Nephrology, ChuiYangLiu Hospital Affiliated to Tsinghua University, Beijing 100022, China; 15 Westlake Omics (Hangzhou) Biotechnology Co., Ltd., Hangzhou 310024, China; 16 Department of Dermatology, the First Affiliated Hospital with Nanjing Medical University, Jiangsu Province Hospital, Nanjing 210029, China; 17 Nanjing University of Chinese Medicine, Nanjing 210023, China; 18 Biomedical Research Core Facilities Platform, Westlake University, Hangzhou 310024, China; 19 Department of Nephrology, the First People’s Hospital of Lianyungang, Lianyungang 222002, China; 20 Department of International Cooperation, the First Affiliated Hospital with Nanjing Medical University, Jiangsu Province Hospital, Nanjing 210029, China; 21 Academy of Clinical and Translational Research, the First Affiliated Hospital with Nanjing Medical University, Jiangsu Province Hospital, Nanjing 210029, China; 22 Department of Information, the First Affiliated Hospital with Nanjing Medical University, Jiangsu Province Hospital, Nanjing 210029, China; 23 Division of Endocrinology, Department of Geriatrics, the First Affiliated Hospital with Nanjing Medical University, Jiangsu Province Hospital, Nanjing 210029, China; 24 Jiangsu Provincial Key Laboratory of Biological Therapy for Organ Failure, Nanjing Medical University, Nanjing 211166, China

**Keywords:** Health sciences, Medicine, Proteomics

## Abstract

Calciphylaxis (calcific uremic arteriolopathy, CUA) is a rare, fatal disorder primarily affecting chronic kidney disease patients, characterized by microvascular calcification, thrombosis, and skin necrosis. In a discovery cohort (3 CUA, 10 uremic), plasma proteomics identified Thrombospondin-1 (THBS1) as the top upregulated hub in CUA, significantly reduced after human amnion-derived mesenchymal stem cell (hAMSC) therapy, alongside latent TGF-β binding protein 1, both linked to coagulation and wound healing. *In vitro* proteomics indicated that THBS1/TGF-β1 blockade impaired CUA serum-induced endothelial adhesion and coagulation. ELISA in combined discovery and validation cohorts (8 CUA, 20 uremic) confirmed this reduction post-treatment (6 patients), independent of systemic inflammation. Multiplex immunofluorescence revealed THBS1 and CD47 co-localized with CD31 and integrin β3 in injured microvessels. A human microvascular chip showed that THBS1 inhibition or hAMSC-conditioned medium alleviates injury. These findings implicate THBS1 as a key factor and potential biomarker in calciphylaxis, suggesting hAMSC therapy as a promising mechanism-based approach.

**Video Abstract:**

## Highlights

• THBS1 and TGF-β1 are promising blood biomarkers for calciphylaxis
• hAMSCs reduce blood THBS1/TGF-β1 levels and improve skin lesions in calciphylaxis patients
• THBS1/TGF-β1 signaling underlies microvascular injury and thrombosis in calciphylaxis
• hAMSCs repair microvascular injury in a human chip model of calciphylaxis

## Graphical abstract

**Figure:**
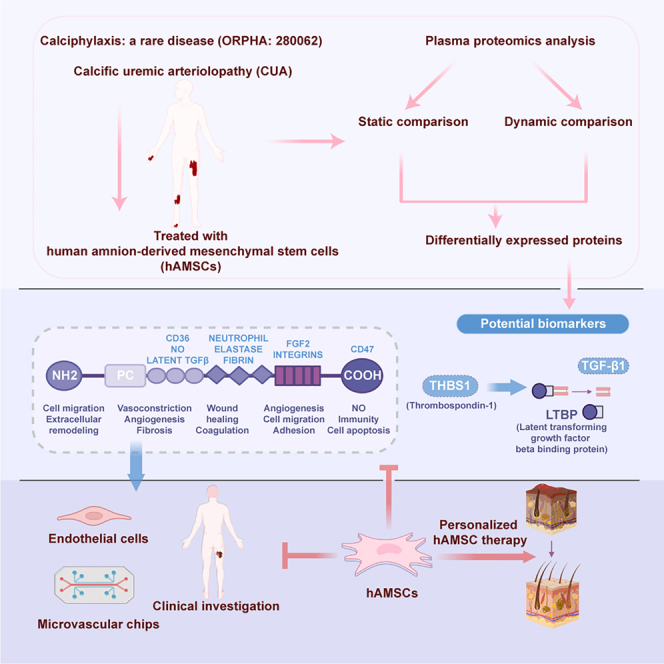

## Introduction

Calciphylaxis, also referred to as calcific uremic arteriolopathy (CUA), is a rare, progressive, ischemic, and painful orphan disease (ORPHA:280062) that predominantly affects patients with chronic kidney disease (CKD). CUA manifests as plaques, nodules, reticulated or gravid purpura, petechiae, and necrotic ulcers with black eschar.^1^ The pathological features include calcification, fibrointimal hyperplasia, and microthrombosis of dermal and subcutaneous arteries and arterioles.^2^ Skin biopsy is the golden standard for definitive diagnosis, but it is invasive. In patients undergoing maintenance hemodialysis (HD), the incidence of calciphylaxis was 3.49 per 1,000 patient-years,^1^ while the 1-year mortality rate is as high as 80% due to sepsis.^3^

To date, there are no approved guidelines for calciphylaxis, and a multidisciplinary approach is required.^4^ Therapeutic options, such as sodium thiosulfate (STS),^3^ bisphosphonates,^4^ anticoagulants, and rheopheresis,^5^ have been reported as effective.^6^ However, recent meta-analyses indicate that STS^7^ and bisphosphonates^8^ do not significantly improve skin lesions or survival in CUA patients compared to other common treatments. In phase 3 trials, SNF472, a specific calcification inhibitor, showed comparable results to the placebo in improving skin lesions and pain in CUA patients.^9^ Cinacalcet treats secondary hyperparathyroidism (SHPT) and may reduce the risk of calciphylaxis, but its impact on disease progression is unclear.^10^ Optimizing the dialysis regimen and surgical debridement are necessary.^4^ Hyperbaric oxygen therapy can be a second-line treatment,^4^^,^^11^ while amputation is a last resort.^12^

Current calciphylaxis studies are mostly case reports or retrospective analyses,^1^^,^^11^^,^^13^^,^^14^ with limited research on potential biomarkers and promising therapies.

Our previous study demonstrated that human amnion-derived mesenchymal stem cell (hAMSC) supernatants contained high levels of hepatocyte growth factor (HGF), angiogenic factors, such as angiopoietin-1 (Ang-1), brain-derived neurotrophic factor (BDNF), as well as moderate levels of vascular endothelial growth factor (VEGF) and fibroblast growth factor 7 (FGF-7), which promote angiogenesis, inhibit vascular calcification, and exert anti-inflammatory and immunomodulatory effects.^15^ hAMSCs and their conditioned medium can promote healing of skin injuries in C57BL/6 mice with deep second-degree burns by inhibiting apoptosis and enhancing cell proliferation.^16^ Our team successfully treated CUA patients with hAMSCs through intravenous and local administration.^15^^,^^17^^,^^18^^,^^19^ Here, we utilized plasma samples from these patients for static and dynamic proteomic analyses to identify core differentially expressed proteins (DEPs), followed by proteomic analysis of human aortic endothelial cells (HAECs) upon intervention targeting the core DEPs pathway. Our objective is to identify non-invasive candidate biomarkers for CUA patients by integrating patient-specific clinical scenario, skin histology, and multiplex immunofluorescence analysis to support clinical translation.

In 1961, Selye et al. introduced the concept of “calciphylaxis”.^20^ Given the complex interplay among uremic toxins, vascular calcification, and microthrombosis in calciphylaxis, accurately recapitulating this human specific disease network in animal models remains challenging.^21^^,^^22^ This study will use a microvascular chip model to explore how potential biomarkers affect calciphylaxis-related vascular injury and to investigate the therapeutic effects of hAMSCs.^23^^,^^24^ Our findings will shed light on biological markers that improve the non-invasiveness and accuracy of diagnosis and guide the optimization of hAMSC treatment regimens for patients with calciphylaxis.

## Results

### Study design

**Figure 1:**
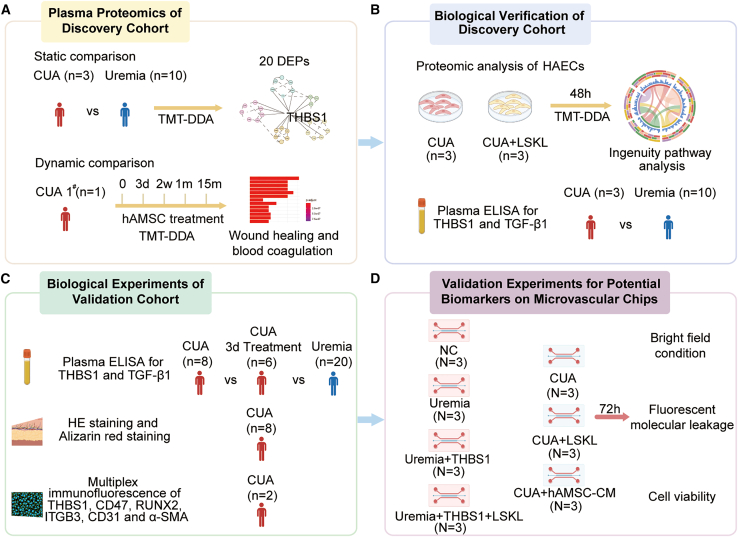
Study design (A) In the discovery cohort, plasma proteomics involved a static analysis of three CUA patients and ten uremia patients, and a dynamic analysis of CUA patient 1 at five time points post-hAMSC treatment. (B) Biological verification within the discovery cohort encompassed cellular proteome sequencing of HAECs stimulated with CUA serum in the presence and absence of LSKL, alongside ELISA assessments of THBS1 and TGF-β1 levels in uremic patients and CUA patients treated with hAMSCs. (C) The independent validation cohort comprised 20 uremia patients and 8 CUA patients, including 6 CUA patients treated with hAMSCs, with further ELISA analysis of THBS1 and TGF-β1 levels, and skin histology and multiplex fluorescence staining linked to core pathway. (D) The damaging effects of THBS1 and hAMSCs’ protective effects were verified on the microvascular chip using groups with uremic serum, CUA serum, THBS1/TGF-β1 pathway inhibitors, and hAMSC-CM stimulation. CUA, calcific uremic arteriolopathy; TMT-DDA, tandem mass tag-data-dependent acquisition; DEP, differentially expressed protein; THBS1, thrombospondin 1; hAMSCs, human amnion-derived mesenchymal stem cells; HAECs, human aortic endothelial cells; ELISA, enzyme-linked immunosorbent assay; TGF-β1, transforming growth factor β1; LSKL, Leu-Ser-Lys-Leu-NH2; ECs, endothelial cells; CD47, cluster of differentiation 47; H&E, hematoxylin and eosin, RUNX2, runt-related transcription factor 2; ITGB3, integrin subunit beta 3; CD31, cluster of differentiation 31; α-SMA, α-smooth muscle actin; NC, negative control; hAMSC-CM, hAMSC-conditioned medium.

The study design is outlined in Figures 1A–1D. In the discovery cohort, we performed in-depth static plasma proteomic analysis in uremic and CUA patients and analyzed dynamic changes in a CUA patient during 15 months of hAMSC treatment at five time points (Figure 1A). Subsequently, we conducted proteome sequencing on HAECs intervened with core DEPs pathway (Figure 1B). In parallel, in both the discovery (Figure 1B) and validation cohorts (Figure 1C), we verified core DEPs levels in plasma during hAMSC treatment using enzyme-linked immunosorbent assay (ELISA). We also investigated histopathological features and target proteins in CUA patient skin tissue treated with hAMSCs (Figure 1C). Finally, using microvascular chips, we validated the damaging effects of the Thrombospondin 1 (THBS1 or Tsp-1)/transforming growth factor β1 (TGF-β1) pathway and the protective effects of hAMSC-conditioned medium (hAMSC-CM) (Figure 1D).

### Baseline characteristics of the discovery and validation cohorts of patients with CUA

In the discovery cohort (Table 1), we found that the CUA group (*n* = 3) showed significantly lower levels of hemoglobin and creatinine, but significantly higher levels of white blood cells, alkaline phosphatase, and hypersensitive C-reactive protein (hs-CRP) compared to the uremic group (*n* = 10). Similarly, in the validation cohort (Table 1), the CUA group (*n* = 8) had longer hemodialysis duration, lower albumin and creatinine levels, and elevated white blood cells, platelet, and hs-CRP levels compared to the uremia group (*n* = 20). Nine CUA patients (3 in the discovery cohort, 6 in the validation cohort), excluding patients 7 and 10, received hAMSC salvage therapy (details in Table S1).

**Table 1:** Baseline characteristics of demographic and laboratory indicators for the discovery cohort and validation cohort

Parameters	Discovery cohort			Validation cohort		
	CUA (*n* = 3)	Uremia (*n* = 10)	*p* value	CUA (*n* = 8)	Uremia (*n* = 20)	*p* value
**Demographics**						

Age (years)	50.67 ± 17.01	51.40 ± 7.49	0.913	47.63 ± 9.93	44.20 ± 7.35	0.323
Male/Female	1/2	4/6	1.000	3/5	10/10	0.686
BMI (kg/m^2^)	18.30 ± 1.93	22.99 ± 3.88	0.074	19.46 (18.69, 20.54)	21.35 (19.78, 23.49)	0.110

**Dialysis vintage (months)**						

HD	72 and 96	31.86 ± 20.40	N/A	158.00 ± 70.96	80.21 ± 57.93	***0.019***
PD	60	33.00 ± 28.62	N/A	84.00 ± 50.91	50.17 ± 33.18	0.302

**Comorbidities, n (%)**						

Diabetic mellitus	1 (33.33)	0 (0.00)	0.231	2 (25.00)	0 (0.00)	0.074
Hypertension	3 (100.00)	10 (100.00)	N/A	8 (100.00)	16 (80.00)	0.295

**Laboratory values**						

Hemoglobin (g/L)	97.67 ± 7.09	112.60 ± 7.59	***0.012***	90.00 (79.50, 103.50)	105.00 (92.50, 115.50)	0.199
White blood cell count (×10^9^/L)	15.96 (13.11, 15.97)	6.00 (3.99, 7.18)	***0.007***	8.91 ± 2.88	6.16 ± 1.47	***0.031***
Platelet count (×10^9^/L)	453.33 ± 206.50	176.90 ± 44.65	0.144	234.13 ± 66.48	187.85 ± 42.74	***0.037***
Albumin (g/L)	36.37 ± 10.51	40.04 ± 4.74	0.610	32.65 ± 2.76	38.29 ± 5.96	***0.017***
ALT (U/L)	14.27 ± 9.09	13.02 ± 5.77	0.776	13.15 (10.00, 23.50)	10.95 (7.25, 16.65)	0.354
AST (U/L)	24.37 ± 17.52	14.24 ± 5.23	0.423	18.85 (12.55, 28.60)	12.45 (9.40, 14.05)	0.099
Creatinine (μmol/L)	473.77 ± 117.70	985.88 ± 384.33	***0.049***	511.89 ± 206.03	1011.16 ± 282.10	***<0.001***
Urea nitrogen (mmol/L)	22.44 ± 5.05	24.49 ± 5.67	0.586	18.85 ± 5.95	20.77 ± 5.98	0.449
Calcium (mmol/L)	2.26 ± 0.28	2.37 ± 0.16	0.413	2.22 (1.91, 2.31)	2.32 (2.17, 2.55)	0.123
Phosphorous (mmol/L)	1.74 ± 0.64	1.86 ± 0.59	0.763	1.80 ± 0.49	2.04 ± 0.56	0.298
iPTH (pg/mL)	317.07 ± 145.73	193.36 ± 78.94	0.073	473.30 (151.95, 785.40)	573.95 (321.10, 680.15)	0.636
ALP (U/L)	141.10 ± 63.35	80.30 ± 22.94	***0.020***	154.50 (109.50, 208.50)	103.50 (60.50, 171.00)	0.099
hs-CRP (mg/L)	50.63 (32.42, 72.52)	4.76 (1.00, 6.32)	***0.014***	93.10 (23.65, 140.90)	4.28 (2.05, 11.12)	***<0.001***

**Pharmacological treatments, n (%)**						

Calcimimetics	1 (33.33)	0 (0.00)	0.231	2 (25.00)	0 (0.00)	0.074
Sodium thiosulfate	2 (66.67)	0 (0.00)	***0.038***	8 (100.00)	0 (0.00)	***<0.001***
Anti-vitamin K anticoagulants	0 (0.00)	0 (0.00)	N/A	0 (0.00)	0 (0.00)	N/A
Bisphosphonates	0 (0.00)	0 (0.00)	N/A	0 (0.00)	0 (0.00)	N/A

BMI, body mass index; HD, hemodialysis; PD, peritoneal dialysis; ALT, alanine aminotransferase; AST, aspartate aminotransferase; iPTH, intact parathyroid hormone; ALP, alkaline phosphatase; hs-CRP, hypersensitive C-reactive protein.

Bold-italic formatting in the *p*-value column denotes statistical significance (*p* < 0.05). This highlights baseline parameters with significant differences between the CUA and uremic groups across both the discovery and validation cohort.

### The core role of THBS1 in the static plasma proteomic network of CUA vs*.* uremic patients

**Figure 2:**
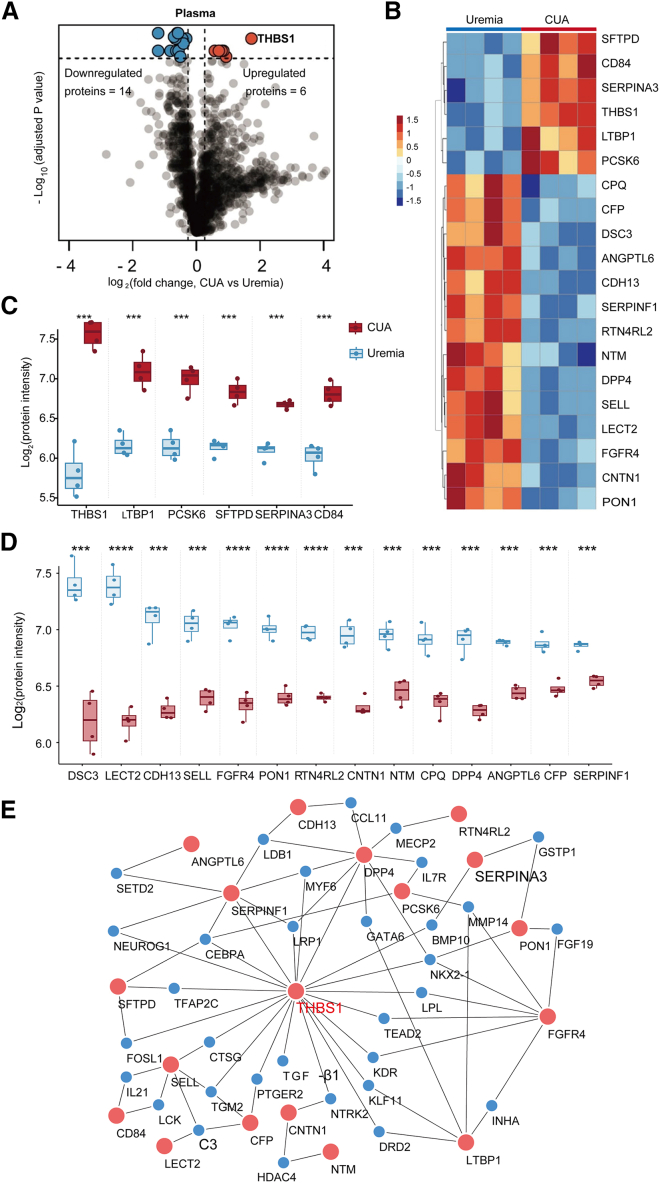
Core role of THBS1 in the plasma DEP network of CUA vs. uremic patients in the discovery cohort (A) Volcano plot of all identified plasma proteins from CUA (*n* = 3 biological replicates) vs. uremic patients (*n* = 10 biological replicates). Points represent individual proteins. Horizontal coordinates show proteins with FC > 1.2 (red) or < −1.2 (blue). The vertical axis displays −log10(*P*) values > 1.30, corresponding to *p* < 0.05 by Welch’s *t* test across two technical replicates. *p* values were adjusted using the Benjamini-Hochberg procedure. A total of 20 DEPs were identified, including six upregulated and 14 downregulated proteins. THBS1 was the most upregulated DEP in CUA. (B) Heatmap of hierarchical clustering of significant DEPs. (C) Expression of upregulated proteins (*n* = 6) in the discovery cohort. (D) Expression of downregulated proteins (*n* = 14) in the discovery cohort. (E) Interaction network of DEPs, with red nodes representing 20 identified DEPs and blue nodes representing connected proteins predicted by IPA. THBS1 is the core protein of the network. THBS1, thrombospondin 1; DEP, differentially expressed protein; CUA, calcific uremic arteriolopathy; FC, fold change; IPA ingenuity pathway analysis. ∗∗∗*p* < 0.001; ∗∗∗∗*p* < 0.0001.

To identify DEPs, we used a tandem mass tag-data-dependent acquisition mass spectrometry (TMT-DDA MS). We identified 1,619 proteins (Figure 2A; Table S2). Through static proteomic analysis of 20 DEPs (Figures 2B–2D) and ingenuity pathway analysis (IPA), we discovered that THBS1 was upregulated in the plasma of the CUA group, with the highest fold change (FC) value among the DEPs (Figure 2C; Table S3). This finding aligns with recent transcriptomic studies showing THBS1 as one of the most upregulated genes in calciphylaxis skin lesions, where it co-localizes with vascular smooth muscle cells.^25^ Notably, THBS1 emerged as the core protein in the DEP network (Figure 2E). Additionally, IPA predicted that THBS1 would inhibit angiogenesis (Table S3).

### Skin regeneration, dynamic core DEP changes in plasma, and pathway enrichment during hAMSC treatment in a CUA patient

**Figure 3:**
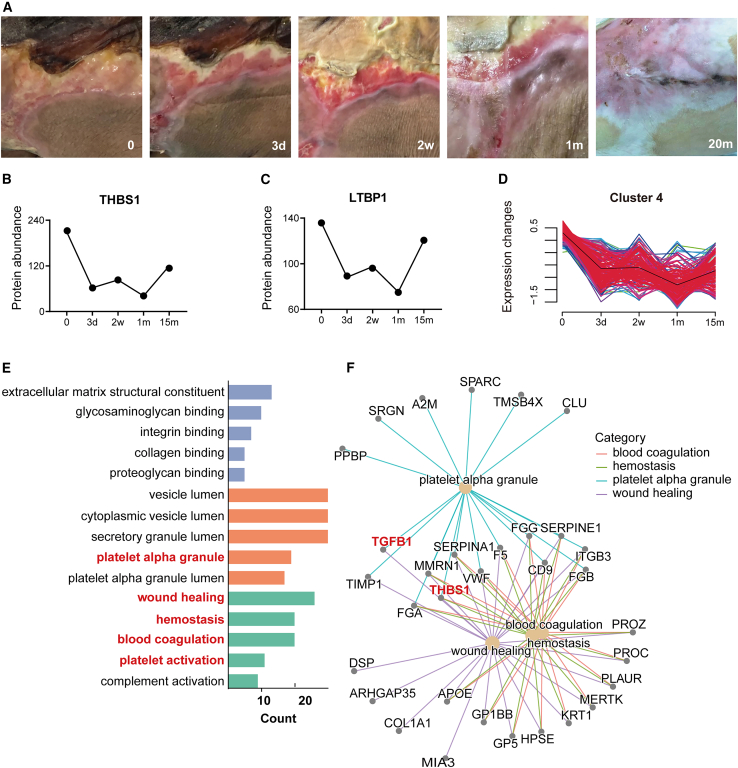
Dynamic characteristics of skin regeneration, core DEPs, and enriched pathways in CUA patient 1 treated with hAMSCs (A) Significant improvement in skin lesions of CUA patient 1 after hAMSC therapy. Before treatment, the left hip showed a necrotizing ulcer with purulent secretion and black eschar. After 3 days of hAMSC treatment, new granulation tissue formed at the wound edges. After 1 month of hAMSC treatment, the epidermis began regenerating, and lesion size reduced. After 20 months of treatment, the skin restructured, forming scar tissue and fully healing. (B and C) Fluctuating reduction in plasma THBS1 (B) and LTBP1 (C) levels in CUA patient 1 was observed after hAMSC treatment. (D) Cluster 4 proteins exhibit trends similar to THBS1 and LTBP1, as analyzed by Mfuzz. (E) GO analysis of proteins in cluster 4. Significant terms include “wound healing”, “hemostasis”, “blood coagulation”, and “platelet alpha granule”, with enriched proteins such as THBS1 and TGF-β1. (F) In the aforementioned entries and the enriched protein network diagram, THBS1 and TGF-β1 serve as hub molecules. DEP, differentially expressed protein; CUA, calcific uremic arteriolopathy; hAMSCs, human amnion-derived mesenchymal stem cells; THBS1, thrombospondin 1; LTBP1, latent TGF-β binding protein 1; GO, Gene Ontology.

We monitored the clinical response of CUA patient 1, who exhibited wound healing and skin regeneration over a 20-months period of hAMSC treatment (Figure 3A). To assess therapeutic efficacy, the Bates-Jensen Wound Assessment Tool (BWAT-CUA) and a visual analog scale (VAS) for pain assessment were employed,^9^^,^^26^ with results illustrated in Figures S1A and S1B.^27^ Three days after hAMSC treatment, improved skin color surrounding wound and decreased peripheral tissue edema were observed, along with the development of fresh granulation tissue (Figure S1A). However, owing to the severity of the cutaneous lesions, the amount of necrotic tissue remained largely unchanged, and pain relief was not evident at this early stage (Figure S1B). Encouragingly, by 15 months of treatment, both the BWAT-CUA and VAS scores had approached normal ranges (Figures S1A and S1B). We monitored changes in the levels of 20 DEPs at 3 days, 2 weeks, 1 month, and 15 months after treatment (Figure S1; dynamic proteomics data in Table S4). Notably, plasma THBS1 levels significantly decreased within 3 days of treatment, with latent transforming growth factor β binding protein 1 (LTBP1) exhibiting a similar trend (Figures 3B and 3C). To further elucidate these temporal patterns, we applied Mfuzz to classify all plasma proteins into 8 clusters (Table S5), among which cluster 4 (containing THBS1 and LTBP1) displayed consistent time-dependent expression profiles (Figure 3D). Subsequent Gene Ontology (GO) analysis of cluster 4 revealed enrichment in “wound healing”, “hemostasis”, and “blood coagulation” (biological processes), as well as “platelet alpha granule” (cellular component) (Figure 3E; Table S6), consistent with the observed wound healing and skin regeneration processes (Figure 3A). Furthermore, protein network analysis identified THBS1 and TGF-β1 as hub proteins within these GO terms (Figure 3F).

### Proteomics analysis of human aortic endothelial cells to analyze the role of the THBS1/TGF-β1 pathway in calciphylaxis

**Figure 4:**
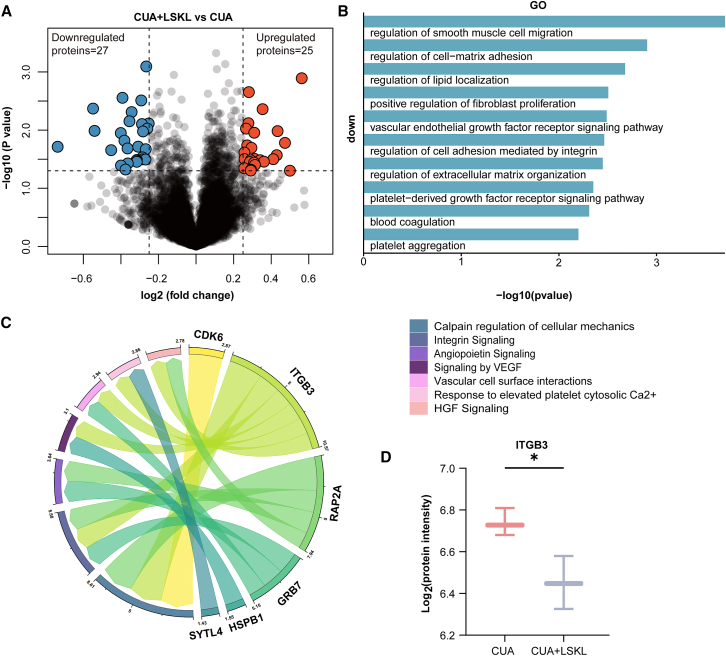
Proteomics analysis of human aortic endothelial cells revealed the effects of inhibiting THBS1/TGF-β1 pathway in CUA within the discovery cohort (A) The volcano plot compares all DEPs identified 48 h after stimulating HAECs with serum from CUA patients + LSKL (*n* = 3 biological replicates) versus serum from CUA patients alone (*n* = 3 biological replicates). Each point on the plot represents an individual protein, with proteins exhibiting a fold change (FC) > 1.2 or < -1.2 and a *p* value < 0.05 by Welch’s *t* test considered differentially expressed. (B) GO analysis by hypergeometric test revealed that LSKL inhibition of THBS1/TGF-β1 enriched downregulated genes in pathways like “smooth muscle cell migration,” “cell adhesion mediated by integrin,” and “blood coagulation”. (C) The chord diagram illustrates the close association of six key DEPs (CDK6, ITGB3, RAP2A, GRB7, HSPB1, and SYTL4), including integrin signaling, angiopoietin signaling and cell surface interactions at the vascular wall. (D) ITGB3 levels in HAECs stimulated with CUA patient serum supplemented with LSKL versus CUA patient serum alone. CUA, calcific uremic arteriolopathy; HAECs, human aortic endothelial cells; DEPs, differentially expressed proteins; LSKL, Leu-Ser-Lys-Leu-NH2; GO, Gene Ontology; THBS1, thrombospondin 1; TGF-β1, transforming growth factor β1; CDK6, cyclin dependent kinase 6; ITGB3, integrin subunit beta 3, RAP2A; Ras-related protein Rap-2a, GRB7; growth factor receptor-bound protein 7; HSPB1, heat shock protein β1; SYTL4, synaptotagmin-like 4. ∗*p* < 0.05.

To investigate the role of THBS1/TGF-β1 signaling in endothelial dysfunction, HAECs were treated with serum from CUA patients in the presence or absence of the THBS1 inhibitor Leu-Ser-Lys-Leu (LSKL, *n* = 3). LSKL is a tetrapeptide derived from latency-associated protein (LAP)-TGF-β that inhibits THBS1-mediated TGF-β1 activation.^28^^,^^29^ Proteomics analysis identified 52 DEPs, with 25 upregulated and 27 downregulated (Figure 4A; Figure S3; HAEC proteomics data in Table S7). GO analysis revealed that the downregulated DEPs following LSKL treatment were significantly enriched in biological processes including cell migration and adhesion, as well as blood coagulation (Figure 4B; Table S8). Additionally, IPA highlighted six key DEPs and their associated biological functions (Figure 4C; Table S9), suggesting a role for the THBS1/TGF-β1 pathway in microvascular injury in CUA. Notably, integrin subunit beta 3 (ITGB3), a critical regulator of endothelial cell adhesion, migration, and proliferation, was significantly downregulated in HAECs treated with CUA serum supplemented with LSKL compared to those treated with CUA serum alone, as demonstrated by quantitative proteomics (Figure 4D).

### Skin pathology staining in the validation cohort

**Figure 5:**
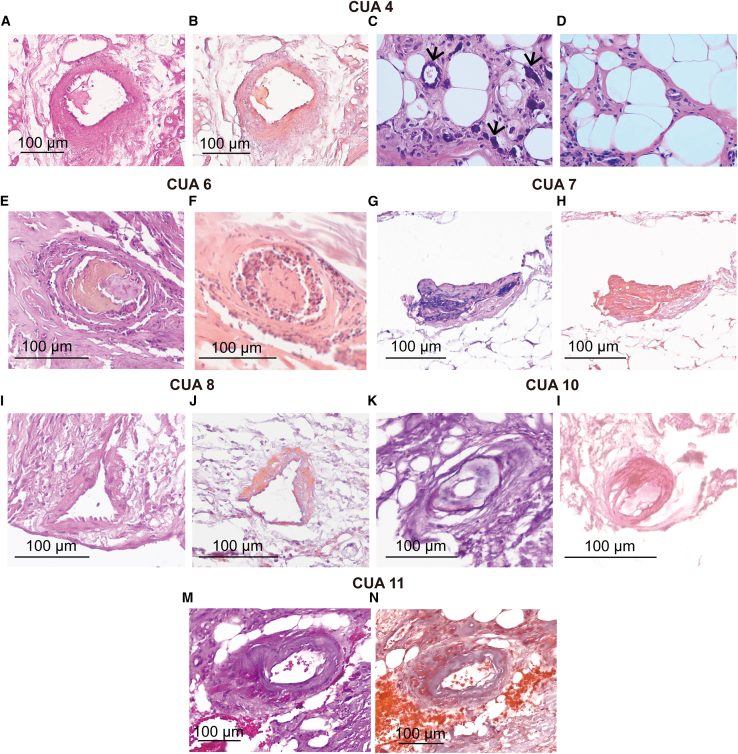
Skin histopathology in calciphylaxis shows microvascular injury and calcification, partially reversed by hAMSC therapy (A and B) Patient 4: H&E staining showing necrosis and thrombosis in microvasculature (A). ARS showing annular calcification and calcium deposition (B). (C and D) Skin samples from CUA patient 4 before and 1 month after hAMSC treatment (H&E staining, × 400). Before treatment, calcium deposition in microvessels and adipose areas (C). Reduced calcium deposition around microvessels and adipose tissue after 1-month hAMSC therapy (D). (E and F) Skin samples from CUA patient 6 showing inflammation, tissue necrosis, and microthrombosis (E, H&E staining) and microvascular calcification (F, ARS). (G and H) Skin samples from CUA patient 7 showing calcification and necrosis in the microvasculature by H&E (G) and ARS (H). (I and J) Skin samples from CUA patient 8 showing inflammation, tissue necrosis, microvascular calcification, and calcium deposition, as detected by H&E (I) and ARS (J). (K and L) Skin samples from CUA patient 10 showing inflammation, swelling, and necrosis of microvessels (K) and microvascular calcification (ARS, L). (M and N) Skin samples from CUA patient 11 showing edema of the vascular wall with perivascular deposition of calcium salts, as detected by H&E (M) and ARS (N). CUA, calcific uremic arteriolopathy; hAMSC, human amnion-derived mesenchymal stem cell; H&E, hematoxylin and eosin; ARS, Alizarin Red S. Scale bars, 100 μm (A–B, E–N).

In the validation cohort, we performed H&E and Alizarin Red S staining on skin tissues from patients in the CUA validation cohort before hAMSC therapy (Figures 5A–5C and 5E–5N). These analyses revealed prominent inflammatory cell infiltration, necrosis, microvascular calcification, and microthrombosis, thereby confirming the initial diagnosis of calciphylaxis in these patients and providing a histological basis for the observed clinical and molecular changes. Figure 5D showed significant histopathological improvement in patient 4 with CUA one month after initiation of hAMSC treatment.

### Plasma THBS1 and TGF-β1 levels measured by ELISA in the discovery and validation cohort

**Figure 6:**
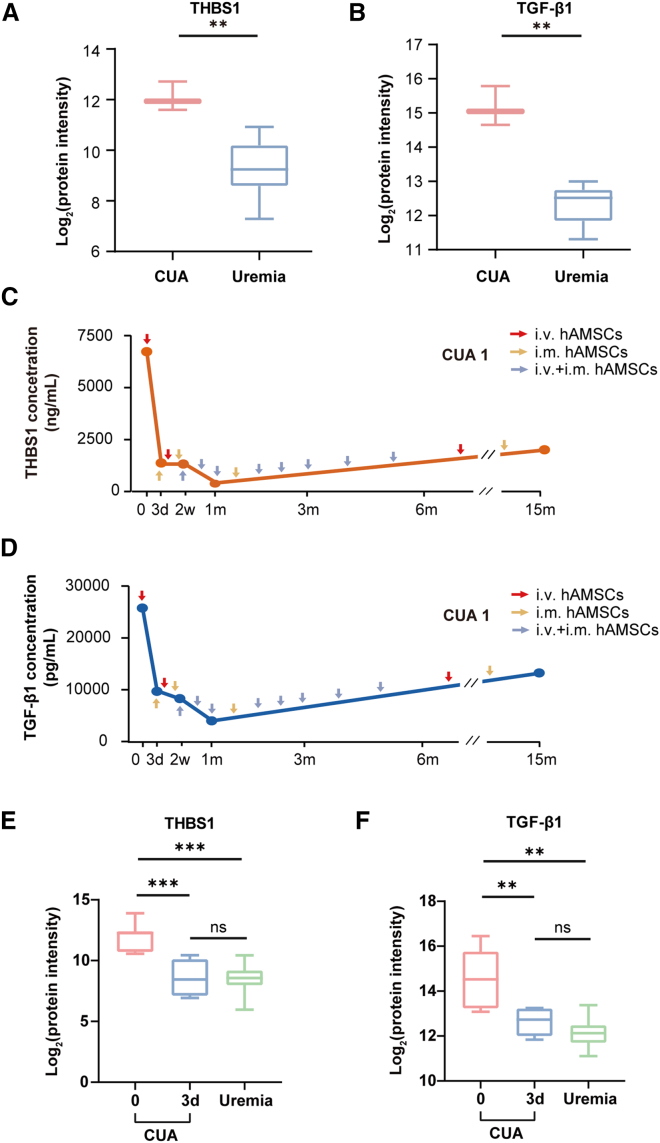
Dynamic plasma THBS1 and TGF-β1 levels monitored by ELISA during hAMSC treatment in discovery and validation cohorts (A and B) Plasma THBS1 (A) and TGF-β1 (B) levels in the discovery cohort (CUA *n* = 3 biological replicates; uremic, *n* = 10 biological replicates). *p* values were calculated using the Mann-Whitney U test. (C and D) Longitudinal profiles of THBS1 (C) and TGF-β1 (D) in CUA patient 1 over a 15-month hAMSC treatment course. (E and F) Plasma levels of THBS1 (E) and TGF-β1 (F) in the validation cohort: uremic controls (*n* = 20 biological replicates), CUA patients pre-treatment (*n* = 8 biological replicates), and 3 days post-hAMSC initiation (*n* = 6 biological replicates). *p* values in (E) and (F) were calculated using one-way ANOVA followed by Tukey’s post-hoc test for homogeneous variance or the Games-Howell test for heterogeneous variances. ELISA, enzyme-linked immunosorbent assay; THBS1, thrombospondin 1; TGF-β1, transforming growth factor β1; CUA, calcific uremic arteriolopathy; hAMSC, human amnion-derived mesenchymal stem cell; i.v., intravenous; i.m., intramuscular. ∗∗*p* < 0.01; ∗∗∗*p* < 0.001.

To quantify baseline differences, plasma levels were measured before hAMSC treatment in the discovery cohort. In the CUA cohort (*n* = 3), data are presented as median (range) due to limited sample size. THBS1 levels were 606.00 (400.78–1177.25) ng/mL in uremic patients and 3912.78 (range: 3103.77–6726.56) ng/mL in CUA patients, while TGF-β1 levels were 5876.00 (3679.50–6906.75) pg/mL and 33859.33 (range: 25761.56–56485.90) pg/mL, respectively (Figures 6A and 6B). The dynamic response to treatment was subsequently evaluated. Initial intensive hAMSC treatment led to a rapid reduction in both candidate biomarker levels. Notably, in CUA patient 1, the levels remained low over 15 months but increased as the treatment frequency decreased (Figures 6C and 6D).

In the independent validation cohort, plasma THBS1 and TGF-β1 levels were significantly lower in both the uremia group and post-treatment CUA group compared to pre-treatment CUA patients (Figures 6E and 6F).

Consistent with these potential biomarker changes, the BWAT-CUA score (Figure S4A) demonstrated improvements in exudate characteristics, exudate amount, peripheral edema, and granulation tissue following three days of hAMSC therapy; however, pain and the amount of necrotic tissue showed no significant relief, attributable to the severity of the cutaneous wounds (Figure S4B). Importantly, ELISA showed 6.72-fold and 3.92-fold reductions in THBS1 and TGF-β1, respectively, 3 days after hAMSC treatment (Figures S4C and 4D), with no significant change in hs-CRP (Figure S5), indicating a specific therapeutic effect rather than a non-specific anti-inflammatory response.

### Multiplex immunofluorescence analysis indicated that THBS1 is involved in CUA microvascular damage

**Figure 7:**
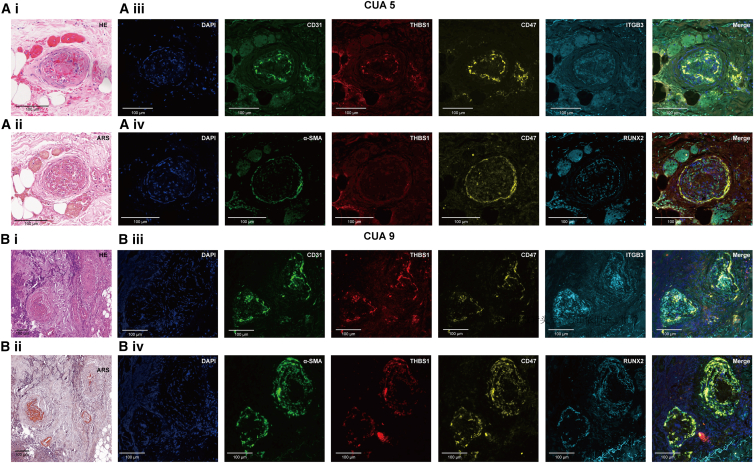
Analysis of skin pathology and THBS1 pathway-related proteins in CUA validation cohort patients (A) Histological and multiplex immunofluorescence analysis of skin tissue from patient 5 with CUA. H&E (AI) and Alizarin Red S staining (AII) showing microthrombi in small arteries. (AIII) Multiplex immunofluorescence for THBS1 and endothelial function-related proteins: DAPI, CD31, THBS1, CD47, and ITGB3, and merged images. (AIV) Multiplex immunofluorescence for THBS1 and vascular smooth muscle calcification-related proteins: DAPI, α-SMA, THBS1, CD47, RUNX2, and merged images. (B) Histological and multiplex immunofluorescence analysis of skin from Patient 9 with CUA. H&E staining (BI) showing thrombus formation in capillaries and endothelial hyperplasia. Alizarin Red S staining (BII) showing annular calcification and calcium salts deposition in thrombi. (BIII) Multiplex immunofluorescence for THBS1 and endothelial cell function-related proteins: DAPI, CD31, THBS1, CD47, ITGB3, and merged images. (BIV) Multiplex immunofluorescence for THBS1 and vascular smooth muscle calcification-related proteins: DAPI, α-SMA, THBS1, CD47, RUNX2, and merged images. CUA, calcific uremic arteriolopathy, THBS1, thrombospondin 1; H&E, hematoxylin-eosin; α-SMA, alpha-smooth muscle actin; CD31, cluster of differentiation 31; CD47, cluster of differentiation 47; ITGB3, integrin subunit beta 3; RUNX2, Runt-related transcription factor 2. Scale bars, 100 μm (A and B).

Multiplex immunofluorescence analysis showed THBS1 co-localized with its receptor CD47 and endothelial markers CD31 and ITGB3 in microvascular thrombosis (Figure 7A) and calcification (Figure 7B) areas of skin biopsies from two representative CUA patients (patients 5 and 9) (Figures 7A and 7B[III–III]). Furthermore, THBS1 and CD47 co-localized with the vascular smooth muscle cell marker α-smooth muscle actin (α-SMA), and with Runt-related transcription factor 2 (RUNX2), a key transcription factor involved in the transdifferentiation of vascular smooth muscle cells into osteoblasts-like cells (Figures 7A and 7B[IV–IV]). These findings suggest that activation of the THBS1 pathway may drive microvascular smooth muscle calcification, endothelial dysfunction, and thrombosis.

### Cell state recording and vascular barrier integrity analysis in the microvascular chip

**Figure 8:**
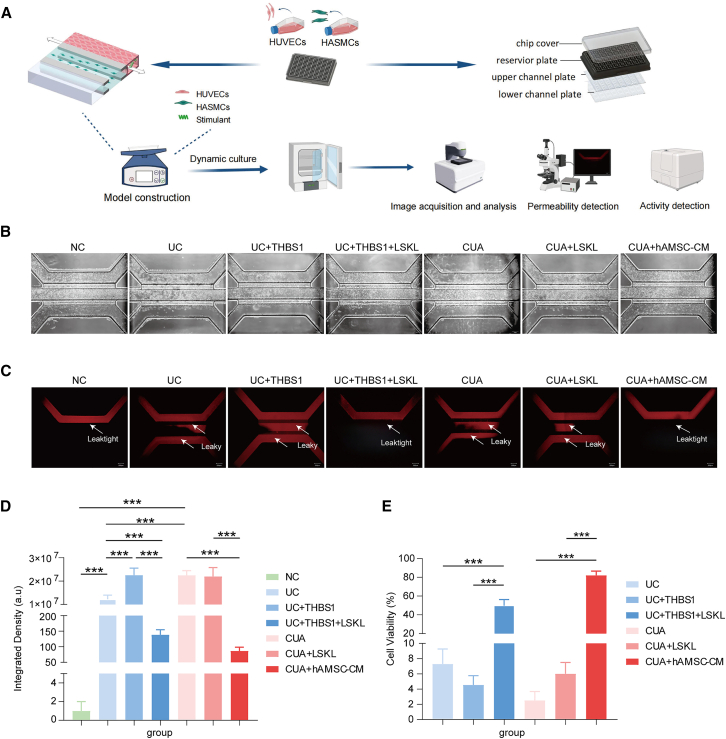
Validation of THBS1/TGF-β1 pathway-mediated damage and hAMSC-CM protection in CUA using a microvascular chip (A) Schematic diagram of organ-on-a-chip model construction and detection of vascular damage caused by serum derived from patients with uremia and CUA. (B) Bright-field imaging showed higher cell viability in uremic serum + THBS1 + LSKL and CUA serum + hAMSC-CM groups after 3 days, with evident cell death in other groups. (C) Fluorescence tracking revealed almost no leakage in the NC group, increased microvascular barrier disruption with THBS1, partial LSKL protection in the uremic serum group but limited protection in the CUA serum group, minimal leakage in the CUA serum + hAMSC-CM group, and significant leakage in other groups. (D) Quantitative integrated density analysis following the addition of fluorescent microparticles (*n* = 3 independent chip units per group; serum pools: CUA, *n* = 4 patients; uremic, *n* = 5 patients). (E) The 72-h viability assay confirmed THBS1-induced damage in the uremia model, with LSKL exerting a protective effect in the uremia group and hAMSC-CM demonstrating superior efficacy in the CUA group (*n* = 3 independent chip units per group; serum pools as in (D)). *p* values in (D) and (E) were calculated using two-way ANOVA followed by Bonferroni’s multiple comparisons test. HUVECs, human umbilical vein endothelial cells; HASMCs, human aortic smooth muscle cells; NC, negative control; THBS1, thrombospondin 1; LSKL, Leu-Ser-Lys-Leu-NH2; CUA, calcific uremic arteriolopathy; hAMSC-CM, hAMSC-conditioned medium. ∗∗∗*p* < 0.001.

To functionally validate our findings, we utilized a microvascular chip model. Figure 8A illustrates the model construction, external appearance, unit structure, and the perfusion system of the microvascular chip. Seven groups were evaluated. After 3 days of dynamic culture (Figure 8B), the negative control and CUA serum + hAMSC-CM groups demonstrated the highest cell viability with no evident cell death. In contrast, the uremic serum + THBS1 + LSKL group exhibited moderate cell survival, whereas substantial cell death was observed in the remaining groups.

To evaluate vascular barrier integrity, 40 μL of 0.5 mg/mL TRITC-labeled dextran (150 kDa) was introduced into the upper channel for fluorescence imaging. Fluorescence tracer tracking revealed minimal microvascular leakage in the negative control group. In contrast, addition of exogenous THBS1 to uremic serum markedly increased vascular permeability, indicating a direct disruptive effect on endothelial integrity. While LSKL conferred partial protection in the uremic serum context, its protective effect was attenuated in the CUA serum group, suggesting a more complex, multi-factorial injury milieu in calciphylaxis. Notably, the CUA serum + hAMSC-CM group exhibited minimal leakage, comparable to baseline levels, whereas all other intervention groups showed significant tracer extravasation (Figure 8C). These results demonstrate that THBS1 contributes to endothelial barrier dysfunction, and that hAMSC-derived factors may effectively preserve vascular integrity even under severe pathological conditions.

To quantify leakage, integrated density (ID) was measured in the middle and lower channels (Figure 8D), and the results were consistent with those in Figure 8C. Higher ID indicated greater fluorescent particle leakage. The following ID values were measured: negative control (NC) group, 1.00 ± 1.00; uremic serum group, 12,200,381.67 ± 1,795,750.99; uremic serum + THBS1 group, 22,533,222.33 ± 2,995,773.17; uremic serum + THBS1 + LSKL group, 138.67 ± 15.95; CUA serum group, 22,447,766.00 ± 1,963,476.97; CUA serum + LSKL group, 21,969,074.33 ± 3,798,023.68; and CUA serum + hAMSC-CM group, 86.67 ± 12.22.

### Cell viability analysis in the microvascular chip

Cell viability was assessed using the Cell Titer-Glo 3D assay, with relative viability calculated as (treated/control × 100%) based on luminescence readings. After 3 days, all groups had lower viability than the NC group. The CUA serum + hAMSC-CM group showed markedly preserved endothelial viability (82.19% ± 4.55%), demonstrating potent, broad-spectrum protection. In contrast, LSKL improved viability in the uremic serum + THBS1 context (49.92% ± 6.33%), but conferred only minimal rescue in the CUA serum group (6.02% ± 1.48%), despite a slight increase compared to untreated CUA serum (2.52% ± 1.17%) (Figure 8E). Two-way ANOVA revealed significant differences in endothelial viability among all groups. These results highlight that while THBS1/TGF-β signaling contributes to microvascular injury, targeting this axis alone is insufficient under systemic calciphylaxis pathology; in contrast, hAMSC-CM provides robust, multi-pathway protection, underscoring its therapeutic advantage.

## Discussion

The management of CUA is challenged by the lack of non-invasive diagnostic tools and effective personalized therapies. Despite its name, CUA involves more than vascular calcification, a common finding in CKD, and is instead characterized by complex microvascular pathology.^30^ Emerging evidence suggests that microvascular thrombosis, driven by a state of hypercoagulability, plays a central role in the development of cutaneous ischemia, necrosis, and severe pain.^31^ This evolving understanding highlights the need to shift focus from calcification alone toward targeting thrombotic and endothelial injury mechanisms.

In this study, we conducted static and dynamic plasma proteomic analysis of CUA patients treated with hAMSCs.^32^^,^^33^ Compared to the uremic group, THBS1^34^ was the core upregulated DEP in CUA patients. Plasma THBS1 levels promptly and significantly decreased after hAMSC treatment, and LTBP1 levels showed a similar fluctuating trend. GO analysis revealed terms including “wound healing,” and “platelet alpha granule,” with enriched proteins THBS1 and TGF-β1.

THBS1, a 420-kDa multidomain glycoprotein, is released by activated platelet alpha granules, with physiological levels in healthy adult plasma typically ranging from 20 to 40 ng/mL.^35^ THBS1 is abundant in megakaryocytes and platelets. Under injury and stress, THBS1 can also be produced by innate immune cells,^36^ endothelial cells, and smooth muscle cells.^37^ THBS1 is not cleared by dialysis^38^ and can bind to the cell surface receptor CD47. It inhibits endothelial cell proliferation,^39^ induces apoptosis^40^ and oxidative stress,^41^^,^^42^ and impairs blood flow.^37^ THBS1 promotes thrombosis by activating platelets^43^^,^^44^; and enhancing thrombin generation.^45^ It also disrupts angiogenesis,^46^ amplifies inflammation,^47^ and regulates immune responses.^48^ A key function of THBS1 is the activation of latent TGF-β1, a cytokine that normally circulates as an inactive complex composed of mature TGF-β1 non-covalently bound to the LAP, which is in turn covalently linked to LTBP1 via disulfide bonds.^49^ THBS1 binds to this latent TGF-β complex through its Lys-Arg-Phe-Lys sequence in the type 1 repeats,^50^ inducing activation by stimulating conformational changes.^51^ This activation pathway contributes to processes, such as wound healing, cell proliferation,^52^ inflammation and fibrosis,^53^ linking THBS1 to vascular pathology in calciphylaxis.

Our integrated findings highlight the central role of THBS1/TGF-β1 signaling in the pathogenesis of calciphylaxis and support its potential as biomarkers. Proteomic analysis in HAECs revealed that inhibition of THBS1/TGF-β1 alleviates endothelial injury, with downstream effects on fibrosis, coagulation, and integrin-mediated cell adhesion. These findings align with Cheeley et al., who noted that vascular calcification alone lacks diagnostic specificity in CUA.^54^ Instead, the combination of microthrombosis, intimal hyperplasia, and severe calcification is pathognomonic. Our proteomic data support this triad, showing coordinated upregulation of platelet activation and coagulation-related proteins, indicative of active thrombogenesis rather than passive calcification.

ELISA confirmed significantly higher plasma levels of THBS1 and TGF-β1 in CUA patients compared to uremic controls. Although hAMSC therapy induced an initial rapid reduction in these proteins, their levels gradually increased during follow-up as treatment frequency decreased, underscoring the need for close monitoring, particularly in early stages, to sustain therapeutic efficacy through prompt dose adjustments.

Immunostaining of CUA patient skin tissues showed local activation of the THBS1 pathway associated with microvascular smooth muscle calcification, endothelial dysfunction, and thrombosis. The lack of clinically relevant animal models has hampered calciphylaxis research and therapy development. In July 2025, the National Institutes of Health (NIH) stopped funding grants relying solely on animal testing,^55^ following the U.S. Food and Drug Administration (FDA)’s April 2025 move to phase out such requirements for drugs.^56^ New approach methodologies (NAMs)—including computer modeling, AI, and organs-on-a-chip—offer promising alternatives. In our microvascular chip models, THBS1 exacerbated vascular injury in uremic serum, causing cell necrosis, reduced viability, barrier disruption, and increased permeability. Notably, blocking THBS1/TGF-β1 partially mitigated damage in CUA serum, whereas hAMSC-CM provided robust protection, indicating that hAMSCs exert potent anti-injury effects likely via modulation of this pathway.

The rapid progression and high mortality of calciphylaxis may stem from the pleiotropic effects of THBS1, including TGF-β1 activation, endothelial dysfunction, inflammation, thrombosis, and vascular calcification. In our cohort, THBS1 levels declined rapidly after hAMSC treatment, more markedly and earlier than hs-CRP, despite no changes in anticoagulation, suggesting that its reduction is not secondary to anticoagulant or general anti-inflammatory effects. While hAMSCs may not directly target THBS1, its consistent and early decrease across patients points to a downstream effect of systemic immunomodulation. The dynamic changes in blood support THBS1 as a responsive and sensitive biomarker of therapeutic impact, rather than a passive bystander. These findings position THBS1 as a central node in the pathogenic network driving vascular injury. Maintaining low THBS1 and TGF-β1 levels through optimized, dynamic hAMSC dosing may be critical. Monitoring THBS1 dynamics could thus enable personalized, precision-guided cell therapy in this devastating disease.

### Limitations of the study

First, the heterogeneity of CUA skin lesions and infection status, compounded by the COVID-19 pandemic, led to inconsistent dosages of hAMSC treatment and follow-up care. Second, the rarity of calciphylaxis results in a limited sample size. While small sample sizes are common in rare diseases,^57^^,^^58^ it is crucial to recruit more patients from multiple centers, optimize treatment protocols, and standardize follow-up procedures to enhance the generalizability and credibility of our findings.

In conclusion, this study integrates clinical observations, proteomic profiling, and human microvascular chip models to identify THBS1 and TGF-β1 as potential biomarkers in calciphylaxis. Despite limited patient numbers, multidimensional analysis reveals that acute microvascular damage driving skin necrosis. Intensive hAMSC therapy rapidly reduces plasma THBS1 and TGF-β1 levels, independent of anticoagulation or general inflammation, supporting their potential role as responsive, prognostic biomarkers. These findings advocate for biomarker-guided, precision application of hAMSC-based therapy. Although THBS1 shows promise, its clinical utility warrants validation in future multicenter, prospective studies with larger cohorts.

## Resource availability

### Lead contact

Requests for further information and resources should be directed to and will be fulfilled by the lead contact, Ningning Wang (wangnn@njmu.edu.cn).

### Materials availability

This study did not generate new unique reagents.

### Data and code availability

• The de-identified clinical, laboratory, and microvascular chip data reported in this study have been deposited in Mendeley Data: https://doi.org/10.17632/38m63b98td.1. The proteomic data have been deposited to the ProteomeXchange Consortium (https://proteomecentral.proteomexchange.org) via the iProX partner repository^59^^,^^60^ (ProteomeXchange: PXD078388). Accession codes are also provided in the key resources table.
• No custom code was used in the proteomics analysis. All downstream analyses were performed in R, and the complete analysis scripts have been deposited at Zenodo: https://doi.org/10.5281/zenodo.18676413 and https://doi.org/10.5281/zenodo.18681679. Accession codes are also provided in the key resources table.
• Any additional information required to reanalyze the data reported in this paper is available from the lead contact upon request.

## Acknowledgments

The authors would like to thank all of the study participants and their families, study coordinators, and support staff involved in the rescue of patients with calciphylaxis for making this study possible. The study was supported by the ISN Mentorship Program and the authors thank Professor Marcello Tonelli (10.13039/100008459University of Calgary, Canada) for his helpful comments on the draft of the manuscript. The authors thank Dr Liang Chen, from the School of Medicine at 10.13039/100018928Westlake University, for his guidance in drawing the images. The authors thank Drs Changying Xing, Chun Ouyang, Yanggang Yuan, Li Zhang, Haibin Ren, Hongqing Cui, and Daoxu Wu in the Department of Nephrology at the 10.13039/100031933First Affiliated Hospital with Nanjing Medical University, Jiangsu Province Hospital, for their assistance in the clinical management of patients. The authors also thank Dr Youjia Yu in the Department of Forensic Medicine, School of Basic Medical Sciences, 10.13039/501100007289Nanjing Medical University, for their support in histopathological staining and analysis. The authors thank Drs Renqiu Wang and Min Dong at Department of Nephrology, ChuiYangLiu Hospital Affiliated to 10.13039/501100004147Tsinghua University for helping to rescue the CUA patient. The authors thank Dr Guangquan Xun at Department of Orthopedics, Yingkou Yanghe Hospital for providing blood and skin tissue samples. We thank LetPub (www.letpub.com) for its linguistic assistance during the preparation of this manuscript. The authors also acknowledge Zongwei Shen, Jianning Wang, and Yunfeng Wang for their assistance in preparing the cover image.

This work was funded by Key Projects of Medical Scientific Research Funded by the 10.13039/501100020205Health Commission of Jiangsu Province (K2024005), the 10.13039/100014718National Natural Science Foundation of China (81270408, 81570666), 10.13039/501100012166National Key R&D Program of China (2022YF0608403 and 2021YFA1301600), 10.13039/501100005145Basic Research Program of Jiangsu (BK20243054), Outstanding Young and Middle-Aged Talents Support Program of The First Affiliated Hospital with 10.13039/501100007289Nanjing Medical University (Jiangsu Province Hospital), Clinical Capacity Enhancement Project (JSPH-MA-2023-7), the 10.13039/501100012166National Key Research and Development Program of China (2017YFC1001303), the State Key Laboratory of Reproductive Medicine and Offspring Health Program (SKLRM-K202105), Specialized Diseases Clinical Research Fund of Jiangsu Province Hospital (XB202403), Jiangsu Province Hospital Maternal and Child Health High-Quality Development Research Program (GZL2504), “Pioneer” and “Leading Goose” 10.13039/100022963R&D Program of Zhejiang (2024SSYS0035), Westlake Omics Junior Clinician Support Program 2021, and Jiangsu Provincial Graduate Research and Practice Innovation Program (SJCX25_0797).

## Author contributions

Conceptualization, N.W., T.G., Y.Z., Y.L., and L.Q.; data curation, N.W., J.H., S.L., X.Y., Q.L., and J. Zhang; formal analysis, Y.Z., N.W., T.G., S.L., X.Y., Y.S., J.H., W.G., Z.X., Z. C., and S.X.; funding acquisition, N.W., J.L., T.G., Y.Z., and L.Q.; investigation, N.W., L.Q., S.L., X.Y., J.H., H.W., M.Z., J.W., K.L., L.Z., Y.Y., Q.L., Z.S., D.L., J. Zhu, C.L., Y.L., L.L., X.T., A.B., and B.Z.; methodology, T.G., Y.Z., Y.L., F.C., S.T., Z.C., S.X., Z.G., J.H., and T.X.; project administration, N.W., J.L., L.Q., Y.L., X.W., and N.L.; resources, J.L., L.Q., H.W., Y.C., C.J., X.M., and S.N.; software, Y.S., M.L., G.C., Z.X., and J.H.,; supervision, N.W., T.G., J.L., and Y.L.; visualization, S.L., X.Y., N.W., J.H., Y.S., Q.L., and W.J.; writing and editing, S.L., X.Y., N.W., Y.S., J.H., J. Zhang, Q.L., and Y.Z.; All other authors reviewed the final draft and agreed to publish the manuscript.

## Declaration of interests

The authors declare no competing interests.

## STAR★Methods

### Key resources table

**Table:** 

REAGENT or RESOURCE	SOURCE	IDENTIFIER
**Antibodies**		

THBS1	Abcam	Cat#ab1823; RRID: AB_2201948
ITGB3	Abcam	Cat#ab179473; RRID: AB_2917988
CD47	Abcam	Cat#ab218810; RRID: AB_3083705
α-SMA	Proteintech	Cat#14395-1-ap; RRID: AB_2223009
RUNX2	Abcam	Cat#ab192256; RRID: AB_2713945
CD31	Abcam	Cat#ab182981; RRID: AB_2920881

**Biological samples**		

Plasma samples	The First Affiliated Hospital with Nanjing Medical University, Beijing Chuiyangliu Hospital, Yingkou Yanghe Hospital, Nanjing BenQ Medical Center, Lianyungang Oriental Hospital and the First People’s Hospital of Lianyungang	CUA patients and uremic patients
Skin samples	The First Affiliated Hospital with Nanjing Medical University, Beijing Chuiyangliu Hospital, and Yingkou Yanghe Hospital	CUA patients

**Chemicals, peptides, and recombinant proteins**		

Triethylammonium Bicarbonate Buffer (TEAB)	Sigma-Aldrich	Cat#T7408
Urea	Sigma-Aldrich	Cat#U1250
Thiourea	Sigma-Aldrich	Cat#T8656
Tris (2-carboxyethyl) Phosphine (TCEP)	Adamas-beta	Cat#61820E
Iodoacetamide (IAA)	Sigma-Aldric	Cat#I6125
Trypsin	Hualishi Tech	Cat#HLS TRY001C
Lys-C	Hualishi Tech	Cat#HLS LYS001C
Trifluoroacetic Acid (TFA)	Thermo Fisher Scientific	Cat#85183
Water	Thermo Fisher Scientific	Cat#W6-4
Acetonitrile	Thermo Fisher Scientific	Cat#A955-4
Formic Acid (FA)	Thermo Fisher Scientific	Cat#A117-50
4′,6-Diamidino-2-Phenylindole (DAPI)	HISTOVABIO	Cat#DAPI100
Phosphate-Buffered Saline (PBS) Powder, pH 7.3	ORIGENE	Cat#ZL1-9061
Frozen Section Antigen Retrieval Solution	Solarbio	Cat#C1035
Endogenous Peroxidase Blocking Buffer	Beyotime	Cat#P0100A
LSKL	MedChemExpress	Cat#HY-P0299A
Endothelial Cell Medium	ScienCell	Cat#1001
Smooth Muscle Cell Medium	ScienCell	Cat#1101
Fetal Bovine Serum (FBS)	ScienCell	Cat#0500
Matrigel®Matrix	Corning	Cat#356231
TRITC-Dextran (MW 150000)	YEASEN	Cat#61234ES10
PBS	Solarbio	Cat#P1020
0.25% Trypsin-EDTA (1×)	Gibco	Cat#25200056
TMTpro 16plex Reagents	Thermo Fisher Scientific	Cat#A44520
High-Select™ Top14 Abundant Protein Depletion Resin	Thermo Fisher Scientific	Cat#A36372

**Critical commercial assays**		

Human Thrombospondin-1 Quantikine ELISA Kit	R&D	Cat#DTSP10
Human TGF-beta 1 Quantikine ELISA Kit	R&D	Cat#DB100C
SOLAμ	Thermo Fisher Scientific	Cat#62209-001
Alizarin Red S Solution	Solarbio	Cat#G1452
Cell Titer-Glo® 3D Cell Viability Assay	Promega	Cat#G9683

**Deposited data**		

Proteomic data	This paper	ProteomeXchange: PXD078388
De-identified clinical, laboratory, and microvascular chip data	This paper	Mendeley Data: https://doi.org/10.17632/38m63b98td.1
Analysis scripts	This paper	Zenodo: https://doi.org/10.5281/zenodo.18676413, https://doi.org/10.5281/zenodo.18681679

**Experimental models: Cell lines**		

Human Amnion-Derived Mesenchymal Stem Cells	The First Affiliated Hospital with Nanjing Medical University	Health donors
Human Aortic Endothelial Cell Line	Professor Zhang Hui, Department of Cardiothoracic Surgery, The First Affiliated Hospital with Nanjing Medical University	Health controls
Human Umbilical Vein Endothelial Cells	ScienCell	Cat#8000
Human Aortic Smooth Muscle Cells	Derived from induced pluripotent stem cells (iPSCs); cultured in smooth muscle cell medium (SMCM) (ScienCell, USA)	iPSC-derived smooth muscle cells

**Software and algorithms**		

Proteome Discoverer Version 2.4.1.15	Thermo Fisher Scientific	https://www.thermofisher.com/us/en/home/industrial/mass-spectrometry/liquid-chromatography-mass-spectrometry-lc-ms/lc-ms-software/multi-omics-data-analysis/proteome-discoverer-software.html
Adobe Illustrator CC2021	Adobe Systems Inc.	https://www.adobe.com/products/illustrator.html
Ingenuity Pathway Analysis (version 51963813)	Kramer et al.^61^	https://www.qiagen.com/cn/
ImageJ/Fiji	National Institutes of Health (NIH)	https://imagej.net
GraphPad Prism 9	GraphPad Software	https://www.graphpad.com
Origin 2024	OriginLab Corporation	https://www.originlab.com/
Metascape	Zhou et al.^62^	https://metascape.org/gp/index.html#/main/step1
R version 4.3.2	R Project	https://www.r-project.org
SPSS 22.0	SPSS Inc.	https://www.ibm.com/spss

**Other**		

Orbitrap Exploris 480 MS	Thermo Fisher Scientific	https://www.thermofisher.cn/cn/zh/home/industrial/mass-spectrometry/liquid-chromatography-mass-spectrometry-lc-ms/lc-ms-systems/orbitrap-lc-ms/orbitrap-exploris-mass-spectrometers/orbitrap-exploris-480-mass-spectrometers.html
Microcon Centrifugal Filters	Millipore	Cat#MRCPRT010
Barocycler® NEP2320	Pressure Bio Sciences Inc	Cat#2320-EXT
Nanoflow DIONEX Ultimate 3000 System	Thermo Fisher Scientific	Cat#6041.7901A
XBridge BEH130 C18 Peptide Separation Technology (PST) Column	Waters	Cat#186003625
FAIMS Pro^TM^	Thermo Fisher Scientific	Cat#FMS02-10001
LH-750 Hematology Analyzer	Beckman Coulter	Cat#6605731
UniCel DxI800 Access Immunoassay System	Beckman Coulter	https://www.beckmancoulter.com/en/products/immunoassay/dxi-800
Olympus AU5400 Automatic Biochemical Analyzer	Olympus Corporation	https://www.olympus-global.com/en/news/1999b/nr990907au5400e.html
Immage 800	Beckman Coulter	Cat#A15501
Microplate Reader	Flash	http://www.shanpu2010.com/
Membrane-Free Barrier Chip	AVATARGET	https://www.avatarget.com.cn/Products/11.html
Rocking Perfusion System	AVATARGET	https://www.avatarget.com.cn/cpfwxq/50.html
Organoid/Organ-on-a-Chip Intelligent Imaging Analysis System	AVATARGET	https://www.avatarget.com.cn/cpfwxq/40.html
DFC7000T Microscope Cameras	Leica	https://www.leica-microsystems.com/products/microscope-cameras/p/leica-dfc7000-t/

### Experimental model and study participant details

#### Ethics, consent, and study approval

This study was conducted in accordance with the principles of the Declaration of Helsinki.^63^ Written informed consent was obtained from all participants. The study protocols were approved by the ethics committees of the First Affiliated Hospital with Nanjing Medical University (approval numbers: 2018-QT-001, 2020-QT-01, 2020-QT-09, 2020-SCR-03, 2025-SR-141) and Westlake University (20200828GTN004). All procedures involving human participants were performed in compliance with institutional ethical standards.

#### Participant recruitment

Between September 2018 and April 2025, 11 patients with calciphylaxis were enrolled, including 3 in the discovery cohort and 8 in the validation cohort. Additionally, 30 age- and sex-matched uremic patients without calciphylaxis were recruited as controls. Of these, 10 control samples were used for plasma proteomics in the discovery phase, and 20 for ELISA validation of candidate biomarkers. CUA diagnosis was based on clinical features including severe pain, palpable subcutaneous swellings, solid hard nodules, necrotizing ulcers, or dry gangrene, typically presenting as stellate ulcers with black eschars.^3^ Histopathology confirmed small vessel calcification, microthrombosis, and fibrointimal hyperplasia in dermal arteries.^1^^,^^2^

Inclusion criteria for uremic controls: (1) age 18–70 years; (2) CKD due to chronic glomerulonephritis. Exclusion criteria: (1) history of malignancy, mental illness, severe cardiovascular disease, shock, liver dysfunction, or secondary kidney disease; (2) arteriovenous fistula stenosis, diabetic foot, or related conditions; (3) pregnancy or lactation; (4) participation in other clinical trials within 3 months; (5) refusal to provide informed consent.

All diagnoses and enrollments were confirmed by three senior clinicians, with frequency matching for age and sex between groups.

#### hAMSC preparation and rescue for calciphylaxis patients

hAMSCs were prepared in a Good Manufacturing Practice (GMP)-compliant facility at the State Key Laboratory of Reproductive Medicine and Center of Stem Cell Research and Clinical Practice, the First Affiliated Hospital with Nanjing Medical University, following national guidelines.^64^ hAMSCs were administered intravenously at 1.0 × 10^6^ cells/kg and locally via intramuscular injection along wound margins at 2.0 × 10^4^ cells/cm.^2^^,^^15^^,^^17^^,^^18^

Nine calciphylaxis patients (CUA 1–6, 8–9, 11) received salvage hAMSC therapy after failing conventional treatments, without adjustments to their anticoagulant regimens. Two patients (Cases 7 and 10) did not receive hAMSCs. Treatment efficacy was evaluated using an 8-item modified BWAT-CUA score and VAS for pain.^9^^,^^26^

#### Patient information for the discovery and validation cohort

All patients in both the discovery and validation cohorts were of Han Chinese ethnicity. The discovery cohort included three CUA patients: a 34-year-old female (Patient 1) with rapid lesion progression but near-complete recovery after 15 months of hAMSC therapy; a 69-year-old female with type 2 diabetes (Patient 2) who discontinued hAMSC treatment after one week due to the onset of the COVID-19 pandemic; a 49-year-old male (Patient 3) undergoing hemodialysis after kidney transplantation, who achieved full lower limb wound healing within 3 months of hAMSC treatment.

The validation cohort included eight patients: a 35-year-old diabetic female (Patient 4) achieved skin regeneration and has been followed up for 30 months to date; a 43-year-old female (Patient 5) showed clinical improvement following two administrations of hAMSCs but discontinued further treatment due to geographical constraints; a 41-year-old male (Patient 6) with post-transplant status and improved distal extremity lesions, who has been followed up for 22 months to date; a 59-year-old female patient with type 2 diabetes (Patient 7) exhibiting progressive necrosis and without hAMSC treatment due to geographical reasons; a 63-year-old female (Patient 8) with large ulcers and limited response to 1 month of hAMSC therapy, who subsequently discontinued treatment; a 43-year-old male (Patient 9) exhibited significant improvement in heel ulcers after two months of treatment but discontinued due to geographical constraints; a 47-year-old male (Patient 10) presented with bilateral lower limb, perineal, and finger necrosis and was unable to receive hAMSC treatment for the same reason; a 49-year-old female (Patient 11) exhibited wound healing following 6 months of hAMSC treatment.

### Method details

#### Static and dynamic plasma proteomic analysis of the discovery cohort

Static analyses included samples from CUA patients 1, 2, and 3, and 10 uremic patients. Two samples were collected from CUA patient 1, and one each from patients 2 and 3. Uremic samples were pooled into four groups. Dynamic analyses were conducted on CUA patient 1 at baseline, 3 days, 2 weeks, 1 month, and 15 months post-hAMSC treatment. Plasma samples were processed by removing high-abundance proteins, followed by concentration, lysis, reduction, digestion, and desalting. Peptides were labeled using TMT and analyzed by liquid chromatography-mass spectrometry/mass spectrometry (LC-MS/MS) after fractionation and re-solubilization.^33^ Data were processed using Proteome Discoverer, with functional enrichment of DEPs. Full Details are provided in the Supplemental Information.

#### Proteomic analysis of HAECs intervened via core DEP pathway

HAECs were starved for 12 h at 80% confluence and then treated with experimental medium containing 3% serum from calciphylaxis patients for 48 h. To assess the role of THBS1, the THBS1 antagonist peptide Leu-Ser-Lys-Leu was used. Each sample contained over 2 × 10^5^ live cells (*n* = 3). Detailed HAEC culture and sample preparation methods for proteomic analysis are provided in the Supplemental Material.

#### Skin histopathological analysis

Skin tissue from CUA patient 4 (pre- and on-treatment) and from the remainder of the validation cohort (pre-treatment) were acquired via deep incisional wedge biopsy,^18^ with written informed consent and institutional ethics approval, in accordance with the Declaration of Helsinki. Sections were stained with hematoxylin-eosin (HE) and Alizarin Red S.

#### Multiplex immunofluorescence

Multiplex immunofluorescence staining was performed on skin tissue sections from CUA patients 5 and 9, prior to hAMSC treatment, using the tyramide signal amplification (TSA) method. The primary antibodies used were anti-THBS1, anti-CD47, anti-CD31, anti-ITGB3, anti-α-SMA, and anti-RUNX2. The staining sequence for endothelial cell-related proteins was CD31, THBS1, ITGB3, and CD47. For vascular smooth muscle cell-related proteins, it was α-SMA, THBS1, RUNX2, and CD47. After deparaffinization and rehydration, paraffin sections were treated with antigen retrieval solution for epitope retrieval at 37°C for 30 min. Sections were then blocked with an endogenous peroxidase blocking solution and washed with phosphate-buffered saline. Tissues were incubated with primary and secondary antibodies, followed by the Dendronfluor E-TSA multicolor fluorescence reagent. The steps of antigen retrieval, blocking, antibody incubation, and TSA were repeated for each target antibody in the specified sequence. Nuclei were counterstained with DAPI, and images were acquired using a microscope-mounted camera (DFC7000T, Leica, Weztlar, Germany).

#### Validation experiments based on a human microvascular chip model

To validate the detrimental effects of core DEPs and the protective efficacy of hAMSC-CM, we established a microvascular chip model comprising seven experimental groups, with three independent chip units per group. Group 1: NC; Group 2: 3% uremic serum (Uremia); Group 3: 3% uremic serum +10 μg/mL THBS1 (Uremia + THBS1); Group 4: 3% uremic serum +10 μg/mL THBS1 + 1% LSKL (Uremia + THBS1 + LSKL); Group 5: 3% CUA serum (CUA); Group 6: 3% CUA serum +1% LSKL (CUA + LSKL); Group 7: 3% CUA serum +10% hAMSC-CM (CUA + hAMSC-CM). The uremic serum was pooled from five patients with uremia, and the CUA serum was pooled from four patients with calciphylaxis.

The microvascular chip was fabricated as follows: A 1.5 μL Matrigel (Corning, USA) suspension containing 1 × 10^7^ human aortic smooth muscle cells (HASMCs)/mL was loaded into the central channel of a membrane-free barrier chip (AVATARGET, China) and gelled at 37°C for 30 min. Then, 2 μL of human umbilical vein endothelial cells (HUVECs) at the same density were added to the upper channel. After 1 h incubation at 37°C, 40 μL of culture medium was added to each reservoir, and the chip was placed on a rocking perfusion system (AVATARGET, China) under 37°C and 5% CO_2_. Medium was refreshed daily, and a mature vascular barrier typically formed by day 5–7.

For sample loading and functional assessment, the liquid was removed from the upper channel of each chip in the experimental groups. Prepare test samples at appropriate working concentrations in culture medium and add 40 μL per well to both reservoirs of the upper channel.

For vascular barrier integrity assessment, remove the liquid from the upper channel of each chip in the experimental groups. Add 40 μL of 0.5 mg/mL TRITC-dextran (150 kDa; YEASEN, China) to the reservoirs on both sides of the upper channel. Immediately use the Organoid/Organ-on-a-Chip Intelligent Imaging Analysis System (AVATARGET, China) to capture 4 × fluorescent images and record the leakage of TRITC-Dextran. Additionally, capture bright-field images of the cells at 10× magnifications on days 0, and 3 for morphological analysis.

Detailed methods regarding ethics approval and participant consent, follow-up of CUA patients, blood sample collection and measurement, cell culture, microvascular chip cell viability assays, and other related procedures are provided in the Supplemental Material.

### Quantification and statistical analysis

Measurement data are presented as mean ± SD or median (interquartile range, IQR), and categorical data as proportions or percentages. IQR were computed using Tukey’s hinges. For baseline comparisons, independent *t*-tests were used for normally distributed data, rank-sum tests for non-normal distributions, and chi-square tests for count data. Statistical analysis was conducted using SPSS 22.0.

Volcano plot *p* values were calculated by Welch’s *t* test, and *p* values in enrichment analysis were calculated by hypergeometric test.

Although THBS1 and TGF-β1 levels were normally distributed, non-parametric Mann-Whitney U tests were chosen as the primary comparisons between groups in the discovery cohort, due to the small sample size (*n* = 3 for the CUA group and *n* = 10 for the uremic group). In the validation cohort, intergroup differences among patients with CUA, uremic patients without CUA, and CUA patients three days after hAMSC treatment were assessed using one-way ANOVA, followed by Tukey’s post-hoc test for variables exhibiting homogeneity of variances or the Games-Howell test for those demonstrating heterogeneity of variance. For *in vitro* cell viability assays, three microfluidic chip units were used per experimental condition. Temporal and intergroup differences were evaluated using two-way ANOVA followed by Bonferroni’s multiple comparisons test. Fluorescent particle leakage was quantified via ImageJ and subjected to the same statistical methodology. Data analysis was conducted using SPSS version 22.0, while data visualization was performed with GraphPad Prism 9. Statistical significance was defined as *p* < 0.05.

### Additional resources

ClinicalTrials.gov Registry Entry: Human amniotic-derived mesenchymal stem cell therapy for calciphylaxis (NCT04592640): https://clinicaltrials.gov/study/NCT04592640.

## Supplemental information

Document S1. Figures S1–S5 and Methods S1

Table S1. Description of hAMSC treatment procedures for nine CUA patients

Table S2. Plasma proteomic matrix of uremic and CUA patients in the discovery cohort

Table S3. Twenty differentially expressed plasma proteins and their IPA analysis were compared between CUA and uremic patients in the discovery cohort

Table S4. Plasma proteomic matrix of CUA patient 1 during hAMSC treatment

Table S5. Diverse dynamic changes in plasma protein levels were observed in CUA patient 1 at different time points during hAMSC treatment, clustered by Mfuzz

Table S6. GO analysis of Mfuzz 4 identified a series of plasma proteins with trends similar to THBS1 at different time points during hAMSC treatment in CUA patient 1

Table S7. Proteomic matrix of human aortic endothelial cells stimulated by CUA serum and CUA serum with LSKL

Table S8. GO analysis of downregulated differentially expressed proteins in human aortic endothelial cells stimulated with LSKL under the context of CUA

Table S9. IPA pathways analysis of differentially expressed proteins in human aortic endothelial cells stimulated with LSKL under the context of CUA

Video S1. Video abstract
