# Supplementary material for: Proteomics and human microchips identify Thrombospondin-1 as a potential biomarker for calciphylaxis stem cell therapy
Source: iScience. 2026 Jun 19;29(7):116388. doi: 10.1016/j.isci.2026.116388 (PMC13315452; doi:10.1016/j.isci.2026.116388)
Supplement: Document S1. Figures S1–S5 and Methods S1 [file mmc1.pdf]

iScience, Volume *29*

Supplemental information

Proteomics and human microchips identify Thrombospondin-1 as a potential biomarker for calciphylaxis stem cell therapy

Jiaying Hu (胡佳颖), Shijiu Lu (卢士玖), Lianju Qin (覃莲菊), Yaoting Sun (孙耀庭), Xiaoxue Ye (叶笑雪), Qinyi Lin (林沁怡), Jing Zhang (张静), Ming Zeng (曾鸣), Jingjing Wu (吴晶晶), Kang Liu (刘康), Jingfeng Zhu (朱敬凤), Ling Zhang (张凌), Feng Chen (陈峰), Zaozao Chen (陈早早), Shihui Xu (徐世慧), Zhangzhi Xue (薛张芝), Yongwu Yu (余永武), Lu Li (李璐), Weigang Ge (葛伟刚), Zhongze Gu (顾忠泽), Cui Li (李翠), Zhonglan Su (苏忠兰), Dan Luo (骆丹), Shaowen Tang (唐少文), Xinfang Tang (唐新仿), Wuziyi Ji (纪吴子仪), Anning Bian (卞安宁), Meihua Liao (廖美华), Guicun Fang (方桂村), Xiang Ma (马翔), Song Ning (宁松), Yugui Cui (崔毓桂), Chunyan Jiang (蒋春艳), Huimin Wu (吴慧敏), Baiqiao Zhao (赵栢桥), Xiuqin Wang (汪秀琴), Ningxia Liang (梁宁霞), Tingyu Xu (徐挺玉), Jiayin Liu (刘嘉茵), Yun Liu (刘云), Tiannan Guo (郭天南), Yi Zhu (朱怡), and Ningning Wang (王宁宁)

SUPPLEMENTAL INFORMATION

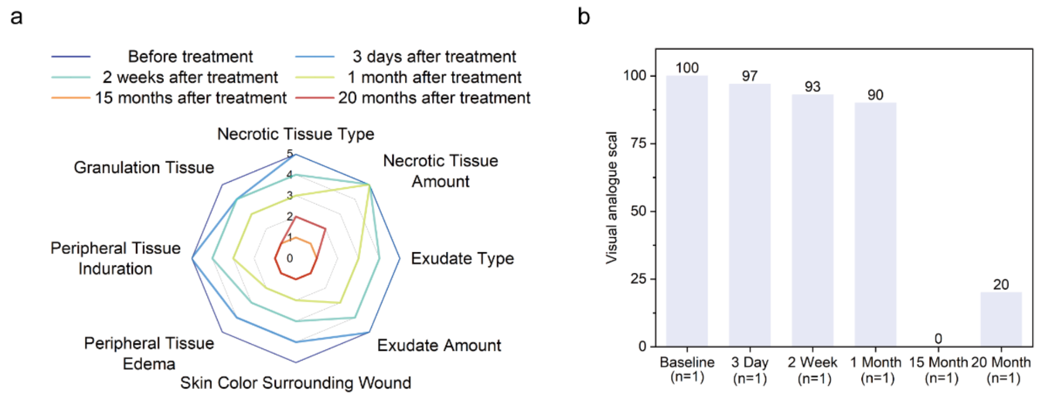

Figure S1. Dynamic changes in BWAT-CUA and VAS pain scores for Patient 1 during hAMSC therapy. (a) BWAT-CUA scores demonstrating progressive improvement in wound characteristics, including enhanced granulation tissue formation and reduced peripheral edema throughout the treatment course. (b) VAS pain scores demonstrating a gradual reduction, approaching baseline normal levels with sustained hAMSC therapy.

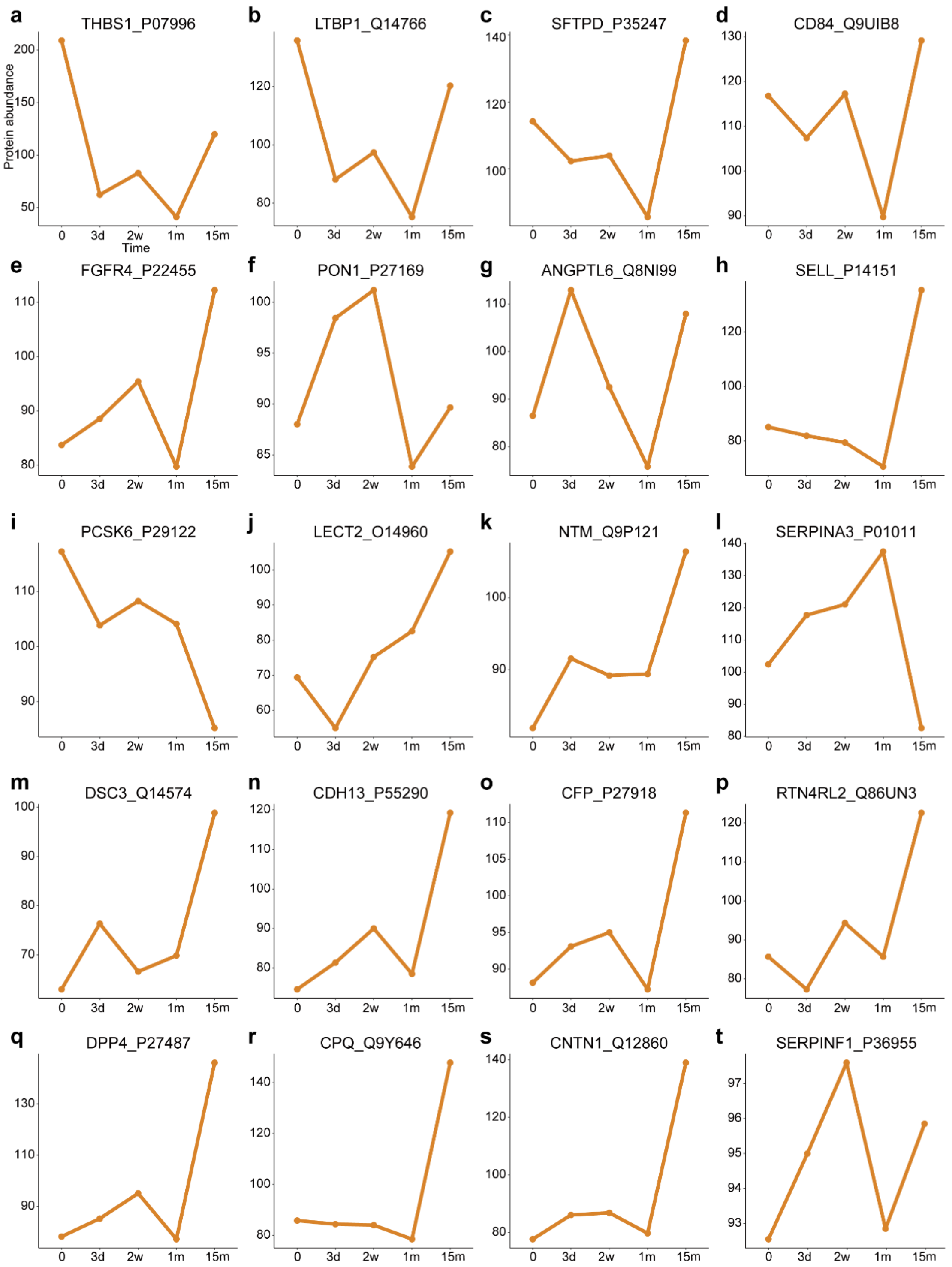

Figure S2. Proteomic analysis of dynamic trends in 20 DEPs in the plasma of CUA patient 1 following hAMSC therapy. (a–t) Individual temporal trajectories of the identified DEPs, comprising six upregulated and fourteen downregulated proteins. Measurements were taken at baseline, 3 days, 2 weeks, 1 month, and 15 months post-treatment.

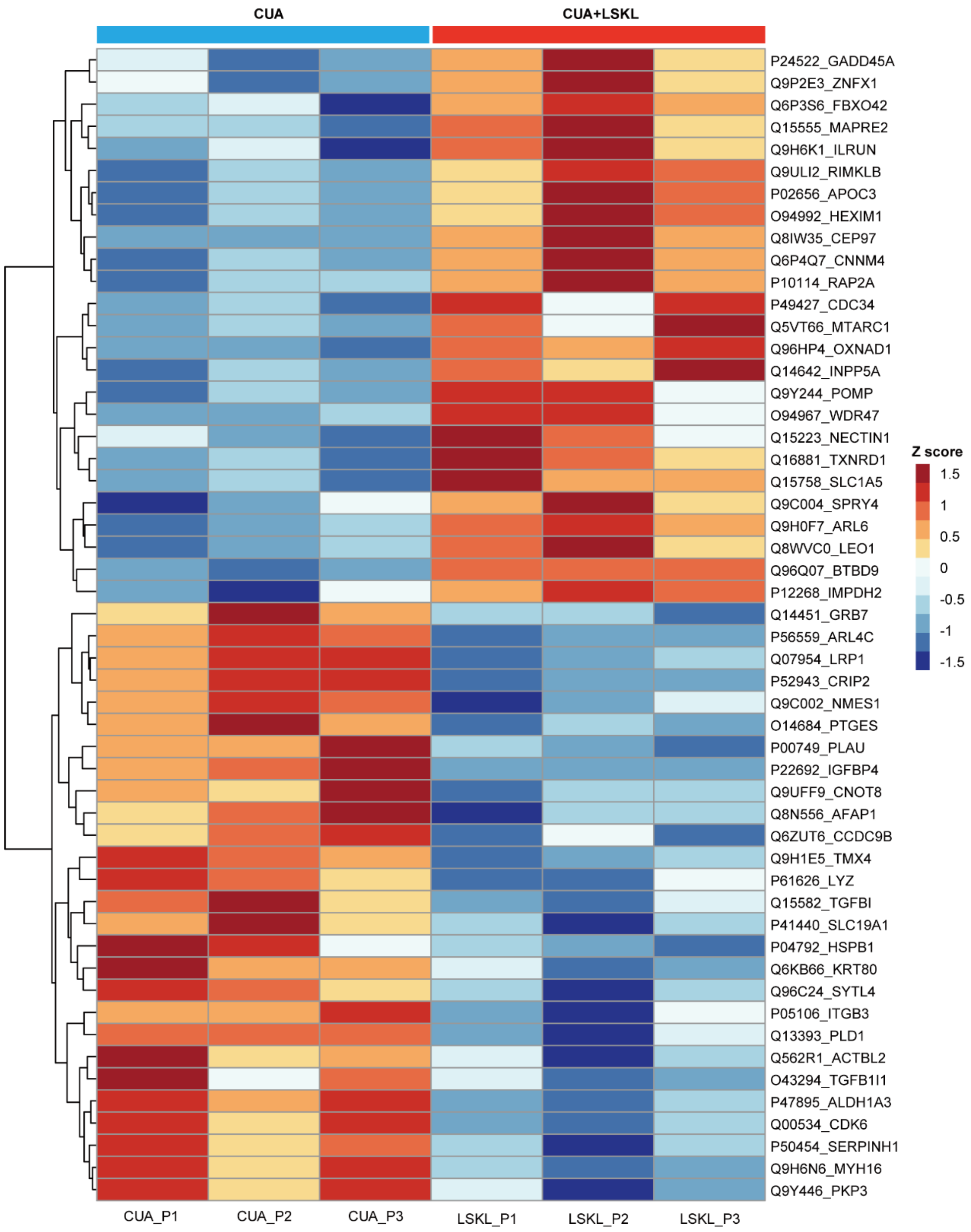

Figure S3. Volcano plot depicting DEPs in human aortic endothelial cell proteomics.

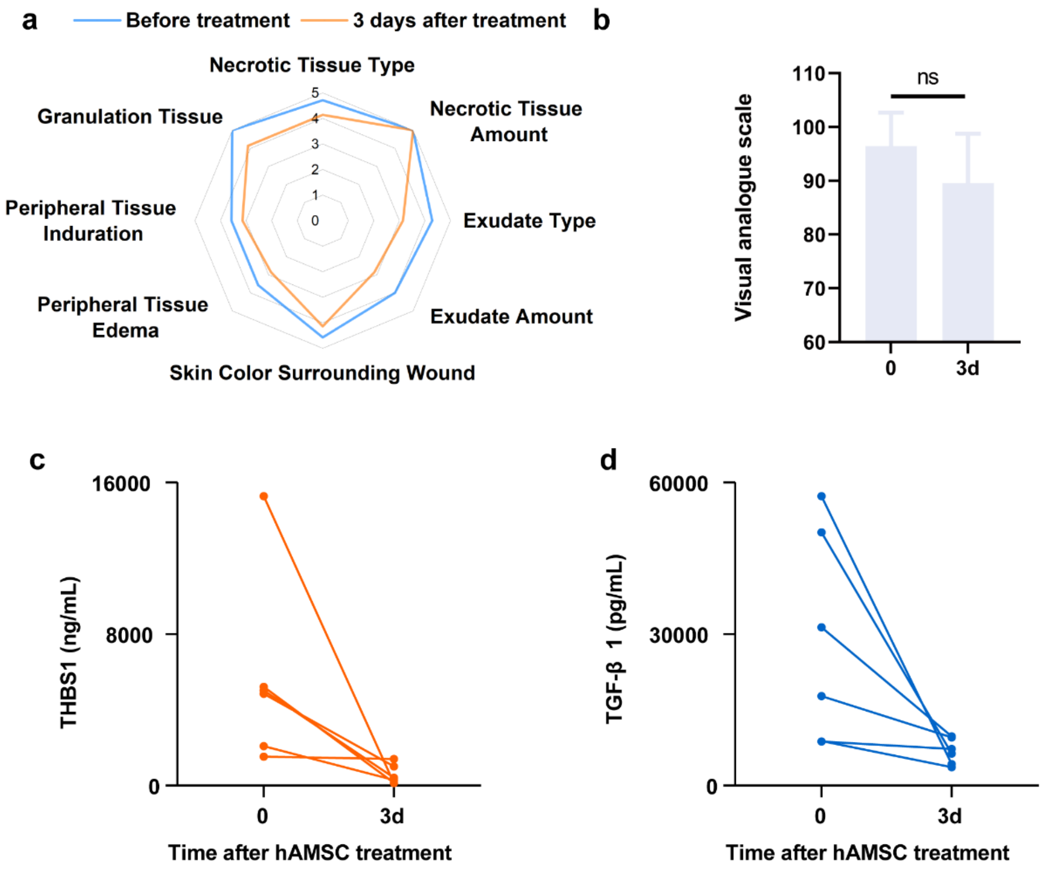

Figure S4. Paired changes in BWAT-CUA, VAS, and plasma THBS1/TGF-β1 levels in six calciphylaxis patients before and after 3 days of hAMSC treatment (validation cohort). (a) BWAT-CUA subscores for exudate characteristics, exudate amount, peripheral edema, and granulation tissue following 3 days of therapy. (b) VAS pain scores and necrotic tissue burden, which remained stable during the initial 3-day post-treatment period. (c) Plasma THBS1 concentrations measured by ELISA at baseline and 3 days post-hAMSC administration. (d) Plasma TGF-β1 concentrations measured by ELISA at baseline and 3 days post-hAMSC administration.

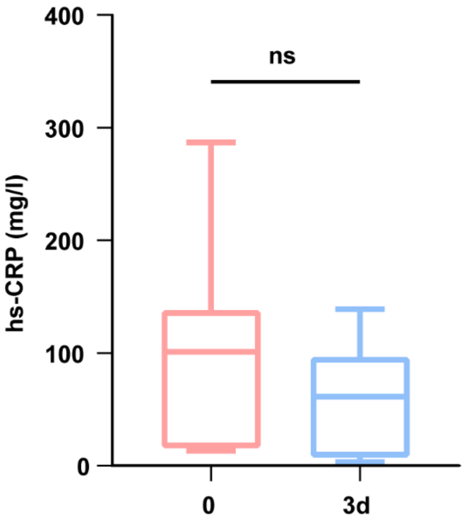

Figure S5. Comparison of hs-CRP levels before and 3 days after hAMSC treatment in the independent validation cohort.

Methods S1. Supplementary Experimental Procedures.

Ethics approval and consent to participate

All participants provided written informed consent, in accordance with the guidelines outlined in the Declaration of Helsinki[S1]. This study was approved by the ethics committee of the First Affiliated Hospital with Nanjing Medical University in China (2018-QT-001, 2020-QT-01, 2020-QT-09, 2020-SCR-03, 2025-SR-141). All procedures carried out in studies involving human participants were conducted in accordance with the ethical standards set out by their respective institutions.

Follow-up of CUA patients

Follow-up began at the start of hAMSC treatment and included clinical visits, telephone and video calls, and online consultation, with a maximum follow-up period of 24 months. Data collection was completed through the electronic medical record system, laboratory tests, and blood and skin pathology analyses. All sample analyses were conducted in the same laboratory to ensure measurement comparability.

Collection and measurement of blood samples

Venous whole blood samples were drawn in the morning after overnight fasting from calciphylaxis and uremic patients and stored at −80°C. Routine blood tests were performed using an LH-750 Hematology Analyzer (Beckman Coulter, Fullerton, CA, USA). Serum biochemical indices were measured using an automatic biochemical analyzer (AU5400; Olympus Corporation, Tokyo, Japan). Serum intact parathyroid hormone (iPTH) levels were measured with a second-generation iPTH assay kit (UniCel DxI800 Access Immunoassay System; Beckman Coulter, Fullerton, CA, USA). Hypersensitive C-reactive protein (hs-CRP) levels were measured using an Immage 800 (Beckman Coulter, Fullerton, CA, USA).

Human aortic endothelial cell culture for proteomic analysis

The human aortic endothelial cell (HAEC) line, provided by Professor Zhang Hui from the Department of Cardiothoracic Surgery at the First Affiliated Hospital with Nanjing Medical University, was cultured in endothelial cell medium (ECM) supplemented with 3% fetal bovine serum (FBS) from ScienCell Research Laboratories (Carlsbad, CA, USA), under a humidified 5% CO_2 atmosphere at 37˚C. The culture medium was replaced every two days.

Protein digestion

Plasma samples

High-abundance proteins in plasma samples were depleted by of High-Select^{TM} Top14 abundant protein depletion resin (Thermo Fisher Scientific). The plasma samples were then transferred to Microcon centrifugal filters with a molecular weight cut-off of 10 kDa for protein concentration. Next, the samples were lysed with a lysis buffer (6 M urea (Sigma-Aldrich, Cat # U1250) and 2 M thiourea (Sigma-Aldrich, Cat # T8656) resolved in triethylammonium bicarbonate buffer (Sigma-Aldrich, Cat # T7408). The proteins were reduced with 10 mM tris (2-carboxyethyl) phosphine (TCEP, Adamas-beta, Cat # 61820E) and alkylated with 40 mM iodoacetamide (IAA, Sigma-Aldrich, Cat # I6125) in darkness[S2]. Furthermore, the proteins were digested with lysine protease (LysC, Hualishi Tech, Cat # HLS LYS001C) for 4 h and with trypsin (Hualishi Tech, Cat # HLS TRY001C) for 8 h, with an enzyme-to-substrate ratio of 1:40 for each step. The reaction was stopped by 10% trifluoroacetic acid (TFA, Thermo Fisher Scientific, Cat # 85183). Peptides were desalted using SOLAμ (Thermo Fisher Scientific, Cat # 62209-001).

Cell sample

Cells were obtained as frozen, non-viable cell pellets after treatment. Sample preparation followed a previously published protocol assisted by pressure cycling technology (PCT)[S3]. Briefly, cell pellets were lysed using lysis buffer, TCEP, and IAA

through a Barocycler® NEP2320 (Pressure Bio Sciences Inc, South Easton, MA) program (30 s 45,000 p.s.i., 10 s 0 p.s.i., 90 cycles, 30°C). Cell lysates were sonicated for 25 s with 1 min intervals on ice, repeated three times. LysC (1/80, w/w) and trypsin digestion (1/20, w/w) were performed using a Barocycler® program (50 s at 20,000 p.s.i., 10 s at 0 p.s.i., 120 cycles, 30°C). The reaction was stopped with 10% TFA, and desalting was performed using SOLAμ C18 columns.

TMT Labeling and Peptide Fractionation

Cleaned peptides were labeled with TMTpro 16plex label reagents (Thermo Fisher Scientific, Cat # A44520). High-pH fractionation was performed using a 120-min gradient on an XBridge BEH130 C18 Peptide Separation Technology (PST) column (300 Å, 5 μm, 4.6 mm × 250 mm, 1/pk; Waters Corporation, MA, USA) connected to a nanoflow DIONEX Ultimate 3000 system (Thermo Fisher Scientific™, San Jose, USA)[S4] The gradient was from 5% to 35% acetonitrile (ACN, Thermo Fisher Scientific, Cat # A955-4) with pH 10.0 at a flow rate of 1 ml per minute, and 60 fractions were collected equidistantly, which were finally combined into 30 fractions.

Mass spectrometry data acquisition

The redissolved peptides were analyzed by liquid chromatography (LC)-MS/MS with the same LC system coupled to an Orbitrap Exploris 480 mass spectrometer (Thermo Fisher Scientific™, San Jose, USA), equipped with a FAIMS Pro™ (Thermo Fisher Scientific™, San Jose, USA) in data-dependent acquisition (DDA) mode.

For each acquisition, peptides were loaded onto a precolumn (3 μm, 100 Å, 20 mm × 75 μm i.d.) using a 60-min LC gradient (from 7% to 30% buffer B) at a flow rate of 300 nl/min (analytical column, 1.9 μm, 120 Å, 150 mm × 75 μm i.d.). Buffer A was 2% ACN with 98% H_2O containing 0.1% formic acid (FA, Thermo Fisher Scientific, Cat # A117-50), and buffer B was 98% ACN with water containing 0.1% FA. All reagents were MS grade. The *m/z* range of MS1 was 375–1800, the resolution at full width at

half maximum (FWHM) was 60,000, the normalized automatic gain control (AGC) target was 300% with an intensity threshold of 2e4, and the maximum ion injection time (max IT) was 50 ms. MS/MS experiments were performed with a resolution at FWHM of 30,000, a normalized AGC target of 200%, and a max IT of 86 ms. The turbo-TMT and advanced Peak Determination were enabled, the isolation window was set to 0.7 Da, and the first mass was set to 110 *m/z*.

Proteomic data processing

The resultant mass spectrometric data were analyzed using Pro DIONEX teome Discoverer (Version 2.4.1.15, Thermo Fisher Scientific, San Jose, USA) against the FASTA downloaded from Human SwissProt on 15 July 2020, containing 20,368 reviewed protein sequences. Protein intensities from a single TMT-16plex experiment were exported for downstream statistical analysis. As all samples were analyzed within a single TMT batch, no additional between-batch normalization was performed. Missing values were rare (0.05% of all quantified data points) and were not systematically associated with any experimental group.

Identification of proteins and specific clusters

From all identified proteins in plasma from uremic calciphylaxis patients ($n = 3$) vs paired non-calciphylaxis patients ($n = 10$), DEPs were selected by Welch's t test and P values were adjusted using the Benjamini–Hochberg procedure (adjusted $p < 0.05$; fold change >1.2 or fold change <−1.2). For longitudinal analysis of plasma proteins in CUA patient 1 after hAMSC treatment, soft clustering was performed using the Mfuzz package in R based on log2-transformed protein abundance profiles. Eight clusters were identified using fuzzy c-means clustering, allowing proteins to exhibit partial cluster membership. THBS1 was assigned to cluster 4.

Pathway analysis

Network pathway analysis tools were used for proteins in cluster 4. The GO processes were enriched by R and visualized as a classical pathway map, enrichment pathways, and a differential protein network relationship map. IPA of the regulated proteins identified the most significant pathways with the P value determined based on a right-tailed Fisher's exact test with the overall activation or inhibition states of enriched pathways predicted by z-score[S5].

Enzyme-linked immunosorbent assay (ELISA)

Collect plasma on ice using EDTA or heparin as an anticoagulant. Centrifuge for 15–20 minutes at 1000 × g within 30 minutes of collection. An additional centrifugation step of the plasma at 10,000 × g for 10 minutes at 2–8 °C is recommended for complete platelet removal. Assay immediately or aliquot and store samples at ≤−20 °C. Avoid repeated freeze-thaw cycles. THBS1 plasma levels were measured in 30 uremic patients, eleven CUA patients before hAMSC treatment, and CUA patients 1, 3, 4, 5, 6, 8,9, and 11 at various time points after hAMSC treatment using the Human Thrombospondin-1 Quantikine ELISA Kit. TGF-β1 in plasma was detected using the Human TGF-β1 Quantikine ELISA Kit, following the manufacturer's instructions. The standards and the samples were analyzed in duplicate.

Deep incisional wedge skin biopsy

Representative lesions were selected for biopsy, avoiding areas that had been excoriated or abraded to ensure preservation of intrinsic pathological features. Deep incisional wedge biopsies were performed under local anesthesia (1% lidocaine). With skin stretched perpendicular to tension lines, a fusiform incision (length:width ≈ 3:1, tip angles ~30°) was made, extending through full-thickness skin into subcutaneous fat to ensure adequate tissue sampling. The specimen was carefully excised without compression to avoid crush artifact and submitted for histopathology. The wound was closed with interrupted sutures after tension release[S6].

Microvascular Chip Experiment

Cell culture

The two-dimensional (2D) cells utilized in this study are adherent cells cultured at 37°C with 5% CO_2. The culture medium is replaced every two days. When the cell confluency reaches 80-90%, subsequent experiments are conducted as required. The culture medium for human umbilical vein endothelial cells (HUVECs) is endothelial cell medium (ECM) (ScienCell, USA). Human aortic smooth muscle cells (HASMCs) are derived from induced pluripotent stem cells (iPSCs) and cultured in smooth muscle cell medium (SMCM) (ScienCell, USA).

Cell viability assay

On day 3 after loading, the Cell Titer-Glo® 3D Cell Viability Assay (Promega, USA) was used for endpoint detection. The chemiluminescent signal was measured using a microplate reader (Flash, China), and the raw luminescence values were normalized to relative viability percentages by calculating the ratio of drug-treated group to control group values multiplied by 100%.

Supplemental References

[S1.] World Medical Association Declaration of Helsinki: ethical principles for medical research involving human subjects. (2013). Jama *310*, 2191–2194. 10.1001/jama.2013.281053.

[S2.] Nie, X., Qian, L., Sun, R., Huang, B., Dong, X., Xiao, Q., Zhang, Q., Lu, T., Yue, L., Chen, S., et al. (2021). Multi-organ proteomic landscape of COVID-19 autopsies. Cell *184*, 775–791.e714. 10.1016/j.cell.2021.01.004.

[S3.] Guo, T., Kouvonen, P., Koh, C.C., Gillet, L.C., Wolski, W.E., Röst, H.L., Rosenberger, G., Collins, B.C., Blum, L.C., Gillessen, S., et al. (2015). Rapid mass spectrometric conversion of tissue biopsy samples into permanent quantitative digital proteome maps. Nat Med *21*, 407–413. 10.1038/nm.3807.

[S4.] Bi, X., Liu, W., Ding, X., Liang, S., Zheng, Y., Zhu, X., Quan, S., Yi, X., Xiang, N., Du, J., et al. (2022). Proteomic and metabolomic profiling of urine uncovers immune responses in patients with COVID-19. Cell Rep *38*, 110271. 10.1016/j.celrep.2021.110271.

[S5.] Krämer, A., Green, J., Pollard, J., Jr., and Tugendreich, S. (2014). Causal analysis approaches in Ingenuity Pathway Analysis. Bioinformatics *30*, 523–530. 10.1093/bioinformatics/btt703.

[S6.] Neitzel, C.D. (2005). Biopsy techniques for skin disease and skin cancer. Oral Maxillofac Surg Clin North Am *17*, 143–146, v. 10.1016/j.coms.2005.02.002.
